# Association between Organochlorine Pesticide Levels in Breast Milk and Their Effects on Female Reproduction in a Taiwanese Population

**DOI:** 10.3390/ijerph15050931

**Published:** 2018-05-07

**Authors:** Men-Wen Chen, Harvey M. Santos, Danielle E. Que, Yan-You Gou, Lemmuel L. Tayo, Yi-Chyun Hsu, Young-Bin Chen, Fu-An Chen, How-Ran Chao, Kuo-Lin Huang

**Affiliations:** 1 Emerging Compounds Research Center, Department of Environmental Science and Engineering, National Pingtung University of Science and Technology, Neipu, Pingtung County 912, Taiwan; 093050cpc@gmail.com (M.-W.C.); power20342000@gmail.com (Y.-Y.G.); hrchao@npust.edu.tw (H.-R.C.); 2 School of Chemical Biological, and materials Engineering and Sciences, Mapúa university, Muralla St., Intramuros, Manila 1002, Philippines; johnharivz@gmail.com (H.M.S.); lemueltayo@yahoo.ca (L.L.T.); 3 Department of Environmental Engineering, National Cheng Kung University, Tainan City 701, Taiwan; aryldiazonium@gmail.com; 4 Department of Environmental Engineering, Kun Shan University, Tainan City 710, Taiwan; ychsu22@yahoo.com.tw; 5 Department of Biological Science and Technology, National Pingtung University of Science and Technology, Neipu, Pingtung County 912, Taiwan; d96642009@gmail.com; 6 Department of Pharmacy & Graduate, Institute of Pharmaceutical Technology, Tajen University, Pingtung 90741, Taiwan; fachen@tajen.edu.tw; 7 Institute of Food Safety Management, National Pingtung University of Science and Technology, Neipu, Pingtung County 912, Taiwan

**Keywords:** organochlorine pesticides, breast milk, breast-feeding, infertility, female reproduction

## Abstract

Only few studies have focused on organochlorine pesticides (OCPs) in breast milk and the related health risks for women in Taiwan. Our goal is to examine breast milk OCPs and their associations with female reproductive function (infertility, gynecological diseases, and menstruation characteristics) as well as their correlation with sociodemographic parameters (age, pre-pregnant body mass index (BMI), annual incomes, population, birth year, and parity) and dietary habit. The breast milk samples were collected in southern Taiwan (*n* = 68) from 2013 to 2016 and the OCP residues were analyzed using high resolution gas chromatography with low resolution mass spectrometry (HRGC/LRMS). The results show that the most abundant OCP residues in the breast milk was ΣDDT with the geometric mean ± standard deviation of 9.81 ± 7.52 ng^−1^ lipid^−1^ followed by ΣHCH (0.539 ± 0.557 ng^−1^·lipid^−1^). In the principal component analysis, *cis-*chlordane (*cis-*CHL) and γ-HCH were found to be related to participants who received medical treatment for infertility, and 4,4′-DDT was associated with those who received gynecological surgery. The logistic regression showed that the odds ratio (OR) of log γ-hexachlorocyclohexane (γ-HCH) was higher for mothers who had received medical treatment for infertility than for the normal group (OR = 25.6, *p* = 0.035) after adjustments for age, pre-pregnant BMI, annual income, population (i.e., native-born Taiwanese), birth year, and parity. Cow milk and beef consumption as well as menstruation characteristics such as average menstrual period (>5 days), shortest menstrual period (<3 days), and women who had taken hormonal drugs were significantly associated to several OCP residues in the breast milk. In addition, ΣHCH including β-HCH and γ-HCH was correlated with annual family income and gravidity as well as cow milk and beef consumptions. Overall, γ-HCH exhibited a probable association with the infertility diseases of Taiwanese women, and dietary habit might play an important role in the female Taiwanese exposure to OCPs.

## 1. Introduction

Organochlorine pesticides (OCPs) are a class of persistent organic pollutants (POPs) that accumulate in the environment and can be distributed through runoffs into surface water or groundwater [1]. OCPs with low estrogenic or anti-estrogenic effects are classified as endocrine disruptors. Lipophilic OCPs, known for having high resistance to degradation as well as long half-lives, can be bioaccumulative in the environment and are usually detected in biological media such as human serum, breast milk, and adipose tissues through everyday diets [2,3]. OCPs have been banned since the 1960s by the Stockholm Convention, but in most developing countries, they are still being utilized illegally [4]. In Taiwan, the government has banned the use of OCPs since 1975. However, the OCPs released before the ban of OCPs use are still present in various environmental and biological media [5,6,7].

OCPs negatively affect human health and have been reported to cause endocrine disruption, reproductive toxicities, neurological disorders, immunotoxicity, and oncogenesis [4]. Meeker, et al. [8] found that serum levels of *p-p*′-dichlorodiphenyldichloroethylene (DDE) cause endocrine disruption by increasing the free thyroxine (T4) and total triiodothyronine (T3) levels and found DDE to be inversely associated with thyroid-stimulating hormone (TSH). Reproductive toxicity studies linking OCPs to female infertility and gynecological issues date back to as early as the 1980s [9]. In an in-vivo study, rats exposed to methoxyclor (200 mg kg^−1^ b.w.) for 7 days showed a reduction in seminal vesicle weight, serum testosterone, and dehydroepiandrosterone levels [10] while mature female rabbits exposed to dichlorodiphenyltrichloroethane (DDT) (3 mg kg^−1^) and lindane (0.8 mg kg^−1^) exhibited reduced ovulation rates [11]. Although several in-vitro studies (e.g., studies using ovarian cells) have listed the negative impacts of DDE and DDT, such as infertility and reductions in the number, development or quality of oocytes, fertilization rate, embryo quality or development, and pregnancy rate, only a few of these studies have managed to obtain statistically significant results [12]. An epidemiological study reported that high levels of serum ΣDDT (DDE + DDT) above 1 μg kg^−1^ were significantly correlated to low pregnancy rates in German women [13]. On the other hand, Axmon, et al. [14] indicated that female serum levels of DDE were not correlated to the prolonged time to pregnancy (TTP) in women living in Greenland, Warsaw, and Kharkiv (*n* = 1722). However, an opposite trend was observed in a Brazilian study that showed that serum DDE from spontaneously pregnant women (fertile group achieving pregnancy in a period no longer than 12 months continuously) was significantly lower in magnitude than those receiving medical treatments for infertility [15]. Infant exposure to OCPs, specifically to DDT and its metabolites through breast milk and maternal serum, has been associated with adverse health effects such as neurodevelopmental delay [16], reproductive dysfunction, preterm, and small-for-gestational-age babies [17]. 

Human breast milk is a convenient specimen that can be used to examine OCP residues in human tissues because it is a non-invasive type of sample [18]. The OCP levels in breast milk reflect maternal burden and can provide information on possible health risks to breastfeeding infants, especially for developing countries. So far, only two studies have reported OCPs in human breastmilk in Taiwan [18,19]. Chao, et al. [18] indicated that DDT and DDE are two dominant OCPs detected in Taiwanese breastmilk. Jaga and Dharmani [20] emphasized that a high DDE/DDT ratio is indicative of high environmental persistence and continuous bioaccumulation while a low DDE/DDT ratio denotes recent DDT exposure. Chao, et al. [18] observed a high DDE/DDT ratio (mean = 13.6, SD = 6.54), which indicated that the OCP residues in Taiwanese breastmilk are the result of past exposure; furthermore, the OCP residue levels in Taiwan have been found to be relatively lower than those of other Asian countries like China, Thailand, Indonesia, and Vietnam but are comparable to those of Japan, Sweden, and the United Kingdom. The levels of OCPs in human breast milk reported by Chao, et al. [18] were comparable to those in human serum measured by Kang, et al. [21], but lower than those in the adipose tissue observed by Quintana, et al. [22]. Like many rapidly developing and industrialized countries, Taiwan is also dealing with environmental health problems caused by OCP contamination. Thus, these compounds should be closely and consistently monitored long-term. However, little attention has been paid to longitudinal study and long-term surveys of OCP exposure to the Taiwanese population because the use of OCPs has been banned by Taiwan’s government for nearly 40 years. Regardless, OCPs are still present in many imported goods in Taiwanese markets and may thus reflect recent exposure. In this study, our goal is to investigate the levels of OCP residues in breast milk samples from Southern Taiwan and to evaluate its health risks, particularly its correlation to female infertility and gynecological diseases.

## 2. Materials and Methods

### 2.1. Sample and Data Collection

The study protocol was reviewed and approved by the Human Ethical Committees of Pingtung Christian Hospital (PCH) in 2013. The study participants were pregnant women without clinical complications recruited from the PCH, Southern Taiwan, from December 2013 to November 2016. The initial selection comprised 143 randomly selected pregnant women ranging in age from 19 to 40 years old. Sixty-eight women were selected based on compliance with the following five criteria: completeness of the questionnaire, native- and nonnative-born populations with minimum residence times of 10 and ≥1 years in Southern Taiwan, respectively (to avoid the inclusion of participants who resided in POP emission areas), no history of PCB poisoning, sufficient breast milk supply (>60 mL), and willingness to strictly follow the protocol to prevent contamination. All participants answered the detailed questionnaires to provide the information for demographic and sociodemographic characteristics, dietary habit, and health or medicine history. The demographic and sociodemographic characteristics were maternal age, pre-pregnant and perinatal body mass index (BMI), parity, population, duration of breastfeeding, educational level, annual family income, and primiparity or multiparity. The dietary habit included the consumptions of cow milk, cheese, egg, beef, pork, and chicken. The self-reported items concerning medicine history such as “Infertility medical treatment” and “Gynecological surgery” were also provided (Table 1 and Table 2). The historical records of OCPs [18,19] (particularly DDT and its metabolites) in Taiwanese breast milk were also included in this study to examine if OCP body burden of women at childbearing age continuously declined after the ban of OCP usage.

### 2.2. Chemical Analysis

In the present study, all of the milk samples were collected from participants who were within the normal duration of pregnancy (36 to 40 weeks), and the milk samples were collected within three weeks after giving birth. The milk samples were collected using chemical-free glass-bottles with Teflon seals and were then frozen in home refrigerators (0–4 °C). After 60–90 mL of breastmilk were collected, the glass bottles were immediately transferred to the laboratory at National Pingtung University of Science and Technology, and the samples were kept frozen at −20 °C prior to OCP analysis. OCP residues in breast milk were analyzed following the protocol from a previous report [18]. The breast milk was homogenized using vertical mixing at 1000 rpm at room temperature for 5 min. Ten microliters of individual internal standards ^13^C_12_-4,4′-DDT and pentachloronitrobenzene, purchased from Cambridge Isotope Laboratories (Andover, MA, USA), were added into a solution containing 2 mL of breast milk, 2 mL of glacial acetic acid, and 2 mL of methanol. The mixture was vortexed and sonicated in an ultrasonic bath for 30 min, and this mixture was moved to a vacuum manifold (Supelco, Oakville, ON, USA) using a 6 cc/5 mg Sep-Pak Vac C_18_ cartridge (Waters, Milford, MA, USA), a 360 mg Sep-Pak Plus NH_2_ cartridge (Waters), and a 3 cc/1 g Bond Elut PCB cartridge (Varian, Harbor City, CA, USA) for solid phase extraction. Twenty different OCPs (EPA method 8081 organochlorine pesticide mixture, AccuStandard, Inc., New Haven, CT, USA) including 4,4′-dichloro-diphenyldichloroethane (DDD), 4,4′-DDE, 4,4′-DDT, α-hexachlorocyclohexane (HCH), β-HCH, γ-HCH, δ-HCH, *cis-*chlordane (CHL), *trans-*CHL, heptachlor, heptachlor epoxide, aldrin, endrin, endrin aldehyde, endrin ketone, dieldrin, decachlorobiphenyl, endosulfan I (a), endosulfan II (b), endosulfan sulfate, and methoxychlor were determined in the breast milk. For the lipid analysis, each breast milk sample was extracted twice using 200 mL acetone/n-hexane (2:1) for 8 min with stirring and filtration. After extraction, the extract was concentrated until dry, and the lipid content was determined using the gravimetric method. The final extracts were analyzed using high resolution gas chromatography equipped with mass spectrometry in the splitless mode (Agilent 7890/5975C-GC/MSD from Hewlett-Packard, Palo Alto, CA, USA) and a capillary column (DB-5MS purchased from J&W Scientific, Folsom, CA, USA) separation in the electron impact (EI) mode. The temperature program of the GC oven increased linearly by 10°C·min^−1^ from 80 °C to 290 °C and was then maintained for 10 min at 290 °C. Two isotopic masses were measured for each mentioned component. Quantification was performed using the isotope dilution method with internal/external standard mixtures. The sample quality control and quality assurance (QA/QC) followed the Taiwan Environmental Protection Agency (EPA) standard method (NIEA T206.21C). The recovery rates of two isotopically labeled standards ranged from 97.4 to 103%. The range of method detection limit (MDL) values, defined as three times the signal-to-noise (S/N) ratio, were from 0.0155–0.0536 ng·g^−1^ ·lipid^−1^ for the twenty OCPs. 

### 2.3. Statistical Analysis

The data for OCPs below MDLs were replaced with their corresponding half MDL (1/2 MDL) values for further statistical analysis. Nonparametric methods, including Mann-Whitney *U* and Kruskal-Wallis *H* tests were used to examine the different groups. The OCP levels were logarithmically transformed to fulfill the normal distribution for statistical analysis. The correlations between the questionnaire parameters (i.e., infertility with medical treatment or never having undergone gynecological surgery) and exposure levels were tested using principal component analysis (PCA) tests. The stepwise linear regression was used to test the correlation of dietary habit and menstrual characteristics against OCP residues in breast milk. Odds ratios (ORs) of mothers’ exposure to OCPs were tested against the parameters of demography or sociodemography, gynecological diseases, menstruation, and dietary habit, using a logistic regression with adjustment variables for pre-pregnant BMI, age, population, annual income, birth year, or parity. In this study, a result was deemed to exhibit statistical significance when it had a *p*-value of ≤0.05. All statistical analyses were performed using Statistical Product and Service Solutions (SPSS), version 12.0 (International Business Machines Corporation, Armonk, NY, United States)

## 3. Results

Table 1 shows the descriptive statistics for the variables in the study. The means and standard deviation (mean ± SD) values for maternal age, pre-pregnant BMI, and parity were 30.5 ± 4.30 years old, 22.5 ± 4.11 kg m^−2^, and 2.21 ± 0.939, respectively. 39.7% of the investigated women were born before 1975. The sample of pregnant women comprised 23.5% primiparous and 77.5% multiparous women. For the population composition, 75% were native-born Taiwanese; 17.6% were native-born aborigines, and 7.4% were nonnative-born Taiwanese. 8.8% of the subjects had reached pre-senior high school levels of education, 39.7% had completed senior high school, 45.6% had attended a tertiary school, and 5.9% had completed graduate school. In addition, the annual income of the pregnant women was also included, with classifications of <$10,000 (14.7%), $10,000–$20,000 (32.4%), $20,000–$34,000 (36.8%), and >$34,000 (16.2%). For the duration of breastfeeding, 2.9% of the pregnant women breastfed their babies less than 2 months. Nine and eight subjects had received medical treatment for infertility and gynecological surgery, respectively. In addition, three participants had an overlap of treatments between infertility and gynecological surgery history. The frequency of the dietary habit and menstruation characteristics in the studying participants are also reported in Table 2.

**Figure 1:**
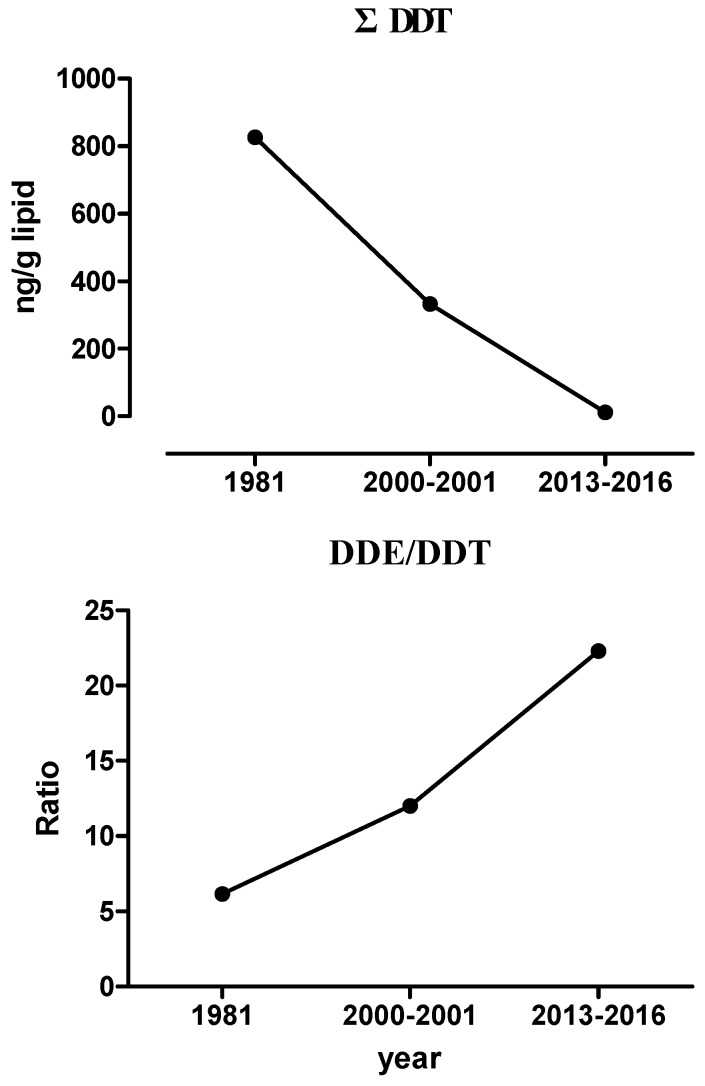
Trends in variations of ΣDDT (sum of DDT, DDD, and DDE) (**upper**) and DDE/DDT ratios (**lower**) in the breastmilk of Taiwanese women in 1981 and during 2000–2001 [18,19] and 2013–2016 (the present study).

Table 3 shows that OCPs were frequently detected in the breast milk samples (detection rate = 85.3% to 100%), with the exceptions of methoxychlor and 4,4′-DDD. The geometric mean concentrations (GM ± SD) of OCPs were observed to be 0.168 ± 0.455 ng·g^−1^·lipid^−1^ for aldrin, 0.839 ± 0.557 ng·g^−1^·lipid^−1^ for ΣHCH (the sum of α, β, γ, and δ-HCH), 0.161 ± 0.284 ng·g^−1^·lipid^−1^ for ΣCHL (the sum of *cis-* and *trans-*CHLs), 0.161 ± 1.64 ng·g^−1^·lipid^−1^ for 4,4′-DDD, 8.07 ± 6.53 ng·g^−1^·lipid^−1^ for 4,4′-DDE, 0.360 ± 0.798 ng·g^−1^·lipid^−1^ for 4,4′-DDT, 9.81 ± 7.52 ng·g^−1^·lipid^−1^ for ΣDDT (the sum of DDD, DDE, and DDT), 0.170 ± 0.500 ng·g^−1^·lipid^−1^ for dieldrin, 0.290 ± 0.776 ng·g^−1^·lipid^−1^ for Σendosulfan (the sum of endosulfan I, II, and sulfate), 0.381 ± 0.701 ng·g^−1^·lipid^−1^ for Σendrin (the sum of endrin aldehyde and ketone), 0.645 ± 0.995 ng·g^−1^·lipid^−1^ for Σheptachlor (the sum of heptachlor and heptachlor epoxide), and 0.0388 ± 0.145 ng·g^−1^·lipid^−1^ for methoxychlor. The highest OCP level was that of 4,4′-DDE, followed by the residue levels of Σheptachlor and ΣHCH in the breast milk of the Taiwanese subjects. The OCP levels of breast milk in this study (Figure 1) were lower than those from 2000 to 2001 reported by Chao, et al. [18] (the levels of the dominant OCPs: *p*,*p*′-DDT (19 ng·g^−1^·lipid^−1^), p,p′-DDE (228 ng·g^−1^·lipid^−1^), α-CHL (7.4 ng·g^−1^·lipid^−1^), heptachlor epoxide (4.0 ng·g^−1^·lipid^−1^), and heptachlor (2.3 ng·g^−1^·lipid^−1^) and the ban of OCP use since 1975 in Taiwan).

A Kruskal-Wallis *H* test was used to examine the significance of OCP levels with regards to pre-pregnant BMI, maternal age, population, parity, and annual income (data not shown). The older mothers (age > 31) had significantly higher *cis-*CHL levels in their breast milk than the younger ones (age ≤ 31) (*p* = 0.043). In addition, the levels of aldrin (*p* = 0.041), 4,4′-DDD (*p* = 0.07), endosufan II (*p* = 0.12), and heptachlor (*p* = 0.06) for mothers who had higher pre-pregnant BMI values were significantly lower than those for mothers who had lower pre-pregnant BMI values. Native-born aborigines had significantly higher Aldrin breast milk levels (*p* = 0.018) and *cis-*CHL (*p* = 0.035) than native-born Chinese mothers. The mothers with annual family income > $34,000 (U.S. dollars) showed significantly lower residual levels of α-HCH and β-HCH than those with incomes <$10,000. In Taiwan, the use of OCPs was banned in 1975, so adjusted variables were used to determine the relation of women’s age to the bioaccumulation of OCPs in their breast milk (Table S1). The Taiwanese women born before the year 1975 had higher concentrations of all OCPs, especially DDE, which is an evident metabolic degradation product of DDT during its long-term presence in the human body. These associations were taken into consideration by using the adjusted variables (i.e., pre-pregnant BMI, age, population, parity, birth year, or annual family income) in the logistic regression. 

The statistical analysis using stepwise linear regressions showed that Log endrin ketone (B = 0.308, *p* = 0.016) had significant correlation to milk consumption while beef consumption was significantly correlated to five OCPs, which included positive correlations to Log endrin ketone (B = 0.284, *p* = 0.011), Log endosulfan sulfate (B = 0.366, *p* = 0.001), and Log *cis-*CHL (B = 0.333, *p* = 0.007) but negative correlations to Log γ-HCH (B = −0.276, *p* = 0.012) and Log endrin (B = −0.278, *p* = 0.016) as shown on Table 4. For the consideration of demographic or sociodemographic characteristics (Table 5), the odds ratio of log endosulfan sulfate (OR = 10.8; 95% CI: 1.03–113; *p* = 0.047) showed a significant association with native-born aborigines compared with those who are native-born and nonnative-born Taiwanese. However, the log concentration of α-HCH (OR = 4.20; 95% CI: 1.08–16.2; *p* = 0.037) or ΣHCH (OR = 10.7; 95% CI: 1.27–90.1; *p* = 0.029) exhibited a significant correlation with the mothers with annual family income ≤20,000 US dollars in comparison to the mothers with annual income >$20,000 U.S. dollar. In addition, compared with the multiparous mothers, ΣHCH (OR = 27.3; 95% CI: 1.63–457; *p* = 0.021) was also significantly associated with mothers under the primiparous group. We didn’t find any significant correlation of OCP residues in breast milk to women’s birth year (before or after 1975), age (over or under 31 years old), pre-pregnant BMI (higher or lower than 21.7 kg·m^−2^), and education level (higher or lower than senior high school) when the logistic regression models were examined with the adjustment of confounders (Tables S2–S8 in the Supplemental Materials). Using the logistic regression model, the dietary habits of the mothers were found to be associated with the risk of OCP residues in their breast milk. These dietary habits included cow milk consumption, cheese consumption, egg consumption, beef consumption, pork consumption, and chicken consumption (Table  and Tables S9–S14). Overall, only cow milk consumption (>625 mL·week^−1^) and beef consumption (>50 g·week^−1^) showed significant values, where significant correlations were found between the log concentrations of β-HCH (OR = 7.35; *p* = 0.016), ΣCHL (OR = 6.65; *p* = 0.039), endosulfan I (OR = 3.03; *p* = 0.043), endosulfan II (OR = 4.53; *p* = 0.005), Σendosulfan (OR = 6.67; *p* = 0.011), endrin (OR = 6.73; *p* = 0.022), endrin ketone (OR = 3.72; *p* = 0.025), Σendrin (OR = 13.3; *p* = 0.007), and Σheptachlor (OR = 9.11; *p* = 0.010) with cow milk consumption, while log concentrations of *trans-*CHL (OR = 5.05; *p* = 0.023), ΣCHL (OR = 10.5; *p* = 0.024), endosulfan II (OR = 4.18; *p* = 0.019), endosulfan sulfate (OR = 7.16; *p* = 0.020), Σendosulfan (OR = 6.88; *p* = 0.027), and methoxychlor (OR = 4.01; *p* = 0.032) in the breast milk were found to be significantly associated with beef consumption. 

As shown in Tables S15–S18, the menstruation characteristics of the mothers, which included mothers who menarche before 13 years old in comparison to after 13 years old, average periods of the menstrual cycle (<28 days), longest period of the menstrual cycle (>37 days), shortest period of the menstrual cycle (≤26 days), average menstrual period (>5 days), longest menstrual period (>7 days), the shortest menstrual period in days (<3 days), and currently taking/have taken hormonal drugs were also correlated with the risk of OCP residues in the breast milk. However, only four of these parameters showed significant associations (Table 5). Log concentrations of *trans-*CHL (OR = 4.73; *p* = 0.035) and endrin ketone (OR = 7.06; *p* = 0.011) were observed to be significantly associated with women whose average menstrual period days was >5 days. Furthermore, log concentrations of *trans-*CHL (OR = 14.9; *p* = 0.020) was also significantly associated with the shortest menstrual period days together with ΣCHL (OR = 14.5; *p* = 0.044). Women who were menstruating fewer than 3 days had a higher risk related to the compounds mentioned. Lastly, significant associations were found in log concentrations of Σendosulfan (OR = 18.6; *p* = 0.035), heptachlor epoxide (isomer B) (OR = 16.6; *p* = 0.015), and Σheptachlor (OR = 21.6; *p* = 0.045) with mothers who had taken or were taking hormonal drugs (Tables S19 and S20). The Odds Ratios (ORs) of OCP residues in the breast milk, which were examined using logistic regression models to account for the breast milk OCP association with disease and those with gynecological surgery history, are summarized in (Tables S21 and S22). Compared with the normal group, the highest OR of γ-HCH was found in mothers who had received infertility treatment (OR = 25.4; 95% CI: 1.26‒519; *p* = 0.035), while no significantly different results between OCPs levels and having undergone gynecological surgery history (*p* > 0.05) were found.

**Figure 2:**
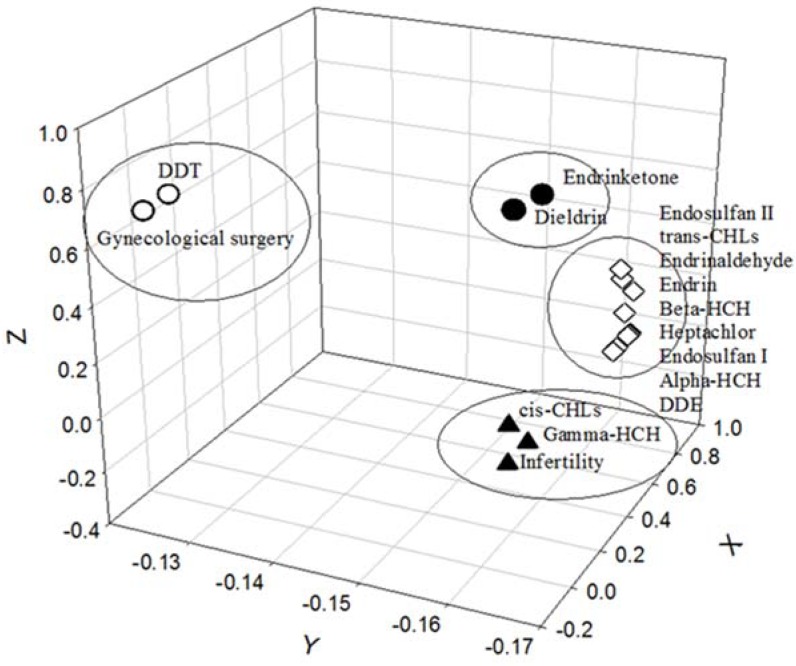
Women who received medical treatment for infertility associated with breast milk *cis-*CHL and γ-HCH or who had undergone gynecological surgery were correlated to breast milk DDT by performing the principal component analysis (PCA).

A principal component analysis (PCA) with a varimax rotation was used to determine the correlations between OCPs in the breast milk samples and participants who had received infertility treatment in obstetric clinics or those who had undergone gynecological surgery at gynecological clinics. Two OCPs, *cis-*CHL and γ-HCH in the breast milk were associated with women having received infertility medical treatment (Figure 2) while 4,4′-DDT in the breast milk was associated with participants who had undergone gynecological surgery.

## 4. Discussion

Figure 1 shows that the ƩDDT declined within the period from 1981 to 2016 while the DDE/DDT ratio increased, signifying the effect of the past usage of this pesticide in Taiwan and its persistence till the present time. As mentioned earlier, a high DDE/DDT ratio is also indicative of the absence of recent exposure. Our median DDE/DDT ratio (22.3) was consistent with that reported by Chao, et al. [18] in Taiwanese human breast milk implying that the OCP levels in the breast milk was from past exposure. Overall, the DDE/DDT ratios from the 1981 [19], 2001–2002 [18], and 2013–2016 (the present study) studies showed an increasing trend reflecting no recent exposure to OCPs among Taiwanese women at childbearing age. However, some research papers from Taiwanese neighboring countries have shown comparable results for the OCP levels in breast milk.

For example, in northern Thailand, the concentrations of heptachlor, heptachlor epoxide, γ-HCH, *p,p*′-DDT, *p,p*′-DDE, and *p,p*′-DDD in 1998 were 4.3, 4.4, 3.6, 69.4, 169.4, and 6.8 ng·mL^−1^, respectively [23]. In China, Wong, et al. [24] reported the OCP levels in breast milk during the period from 1999–2000 to be 0.70, 2.85, and 1.11 μg·g^−1^·lipid^−1^ for *p,p’*-DDT), *p,p’*-DDE, and β-HCH, respectively, while the data from 2003 to 2005 from Zhao, et al. [25] were 1528, 214, 76.2, and 16.7 ng·g^−1^·lipid^−1^ for *p,p*′-DDE, β-HCH, α-HCH, and γ-HCH, respectively. Afterwards, the data in 2006, 2008, and 2010 from Zhou, et al. [26] were 67.1, 25.5, and 10.5 ng·g^−1^·lipid^−1^ for β-HCH, HCB, and 4,4′-DDD (dominant OCPs), respectively. Minh, et al. [27] indicated that the levels of *p,p*′-DDE, *p,p*′-DDT, β-HCH, and *p,p*′-DDD in the breast milk of Vietnamese women from 2000 to 2001 were 420–6300, 34–6900, 11–160, and 3–50 ng·g^−1^·lipid^−1^, respectively. Among the OCPs in breast milk, DDT is still a dominant species in China and Vietnam, while β-HCH and HCB are the most prominent ones for Chinese mothers [28]. Breast milk collected in Korea in 2011 showed OCP levels of 91.7, 20.5, 6.51, 2.22, and 0.94 ng·g^−1^·lipid^−1^ for *p,p*′-DDT, β-HCH, *p,p*′-DDD, heptachlor epoxide, and *p,p*′-DDE, respectively [29]. In other developing Asian countries like India and Saudi Arabia, among all the available commercial products with OCPs, only γ-HCH is permitted to be used for agricultural purposes, which is a possible reason for its higher mean concentrations of 0.37, 0.35, 0.35, and 0.29 mg·L^−1^ in four cities (Al-Hassa, Al-Khobar, Al-Jubail, and Al-Dammam, respectively) where HCH was dominant in the breast milk samples [30,31]. Therefore, this study shows the OCP levels in Taiwan to be lower than those in the neighboring countries (Table 6). Studies by Coakley, et al. [32] and Johnson-Restrepo, et al. [33] showed that high levels of OCPs found in breast milk collected in the USA were higher than that in Taiwan. Some countries such as Russia [34], Colombia [35], and Norway [36] have exhibited higher levels of OCPs in breast milk as compared to our data. The levels of OCPs in breast milk decreased in Taiwan after the ban of OCP use in 1975 based on the OCP data in both our previous study [18] and in the present study (Figure 1). A similar trend has also been observed in American and European countries [37,38,39].

Certain OCPs, especially for HCH isomers, have significant associations with sociodemographic characteristics in the present study. In a Tunisian study [40], levels of hexachlorobenzene (HCB), HCH isomers, and DDT isomers and metabolites in adipose tissue were not significantly related with sociodemographic characteristics such as gender, parity, habitat areas, and smoking habit except for age which showed a positive significant association. For the Tunisian female, the adipose levels of HCB and HCHs had significant correlations with BMI while there were no correlations found between OCPs in adipose tissue and dietary habit. Chao, et al. [18] observed that maternal age had significant associations with γ-HCH in breast milk which was also positively linked to mothers who have high fish consumption. In the present study, cow milk consumption and the shortest menstrual period were slightly and significantly correlated with endrin ketone and *trans-*CHL, respectively, after examining the stepwise linear regression models with adjustment of their confounders. 31.3% linearity of beef consumption could be explained by the predictors of log endosulfan sulfate, Log *cis-*CHL, Log γ-HCH, Log endrin ketone, and Log endrin when the stepwise linear regression model was used. In the stepwise linear regression, dietary habits like cow milk and beef consumption also had significant correlations (positive or negative) to OCPs, indicating that consumption of these foods was a possible exposure pathway for OCPs (Table 4). Compared with the logistic regression model, simple significant outcomes were shown by testing the stepwise linear regression model. In our study, ΣHCH was found to have significant associations to several sociodemographic parameters, particularly annual family income and gravidity in the logistic regression model analysis (Table S23). Some OCP residues in breast milk were significantly and positively correlated with consumption of cow milk or beef (Table 4). Our result showed increased consumption of cow milk and beef increased the risk of being exposed to OCPs, especially to HCH, Heptachlor, and Endrin. Sharma, et al. [41] reported that DDT and HCH (67% for β-HCH contributed) residues were both dominant OCPs in a cow milk sample and that dairy milk intake could be the main source of human exposure to these OCPs. A recent study in China observed that β-HCH was the dominant OCP in beef muscle tissue (mean: 1.18 ng·g^−1^·f.w.^−1^) and cow’s milk (approximately 4.5 ng·g^−1^·lipid^−1^) [42] which is possibly due to still ongoing use of lindane and its persistence and high bioaccumulative potential [43]. In this study, β-HCH was the main residual OCP that is commonly accumulated in food chains and could possibly cause adverse effects on human health. The other recent and immediate sources of Taiwanese exposure to OCPs may be attributed to fish consumption although our results didn’t show any significant evidence (data not shown). Chang [5] found the levels of 20 OCPs to be lower than the OCP MDLs in sediments from Taiwanese aquafarms, indicating no new OCPs contaminants from the land into these aquafarms, but certain OCP residues were detectable in fish samples from both the aquafarms and the sea near the coast. He also examined one of possible sources of OCPs through fish consumption by means of estimated daily intakes (EDIs) and assessed their risks to humans [5]. Bempah and Donkor [44] reported that imported goods such as fruits sold in different markets in Ghana were contaminated with OCPs. Although OCPs have been banned in Taiwan for >40 years, contaminated imported food crops from developing countries are possible sources of OCPs detected in the breast milk of Taiwanese women.

As shown in Figure 2, using the PCA analysis, breast milk DDT was observed to be correlated with women who had undergone gynecological surgery as well as breast milk *cis-*CHL and γ-HCH in women who had received infertility treatments. Several epidemiological studies have indicated negative correlations between semen quality (e.g., low sperm motility) and γ-HCH in semen or serum samples from male adults [45,46,47]. Pant et al. [46] revealed that α-, β-, γ-, δ-HCH and DDT and its metabolites, including the levels of DDD and DDE found in the semen of infertile males were significantly higher than those in fertile men. As compared to reproductive studies conducted on male adults, there have been only a few studies focused on the reproductive effects of γ-HCH exposure on female adults. One example of these female reproductive studies is an American case-control study that examined the ORs of endometriosis risk between serum levels of OCPs in both cases and controls [48]. Upson et al. [48] indicated that higher γ-HCH concentrations were significantly correlated with higher ORs of ovarian endometriosis, but the associations of γ-HCH, heptachlor epoxide, oxychlordane, *trans-*nonachlor, DDE, DDT, and dieldrin did not appear to be significant. Additionally, a hospital-based case and control comparison study indicated that the maternal and cord blood levels of α, β, γ-HCH in the control group were significantly lower than those in the small-for-gestational-age (SGA) case group while there were no significant differences observed for DDE, DDT, DDD, and endosulfan [49]. 

Overall, based on the above-mentioned studies, HCH isomers in human samples seem to be associated with female reproduction disruption. Many studies have also indicated that γ-HCH is the detected dominant OCP residue [48,50,51]. Xu, et al. [52] evaluated the results of the National Health and Nutrition Examination Survey 1999–2004 and found that levels of OCPs like β-HCH, *trans-*nonachlor, and dieldrin in serum were significantly associated with the prevalence of prostate cancer. In an age-period-cohort analysis conducted by Ho, et al. [53] a steady increase in percent-age change in the breast cancer mortality rate was observed with values ranging from 54.8% (aged 20–44 years) to 150% (aged 45–64 years) during the periods of 1971–1975 and 2006–2010. The 1951 birth cohorts showed the greatest mortality risk with peak mortalities in both the 45–49 and 50–54 age groups. The heavy use of DDT to control malaria in Taiwan might be an important reason for heightened breast cancer mortality risk [53]. Airaksinen, et al. [54] indicated that OCPs were correlated to the prevalence of type 2 diabetes. Parkinson’s disease was also correlated to the neurotoxicity caused by the OC pesticide heptachlor [55], and an increased risk was observed in a Costa Rican population with exposure to the occupational pesticide, dieldrin [56]. In addition to these adverse health effects on adults, the exposure of nursing infants to these harmful compounds may cause neurodevelopmental disorders [57], reduced fetal growth [58], endocrine disruption [59], allergies [60], immunotoxicity [61], and oncogenesis [62].

In this study, the logistic regression exhibited high ORs of γ-HCH or lindane in mothers who received infertility treatment as compared to the normal group after adjusting for age, pre-pregnant BMI, annual income, and population (Table S21). Our results infer that the elevated levels of ΣCHL, ΣDDT, ΣHCHs, or other OCPs in breast milk have no significant correlation to women who had undergone gynecological surgery (Table S22). Although the highest OR of γ-HCH was associated with females receiving infertility treatments (OR: 25.4; 95% CI: 1.26‒519), the observed wide range of 95% CI of γ-HCH was probably due to the small sample size (*n* = 68) and small number of infertility cases (*n* = 9) as compared to the control (*n* = 59). Thus, this situation might not be adequate to explain the significantly higher γ-HCH breast milk levels in women receiving infertility treatments as compared to that of the normal women. Exposure to OCPs may delay conception and cause infertility [63]. Previous studies have focused on the association of OCP exposure with both male and female infertility. A review article showed that exposure to DDE, DDT, or HCH resulted in higher ORs of reproductive diseases such as infertility, low sperm count, and low semen volume as well as low counts of oligoospermia, asthenozoospermia, and cryptorchism [64]. Considering the γ-HCH levels in male adults in terms of relevance to semen quality, several reports have observed significantly higher γ-HCH levels in infertile men [45,46,65]. In addition, exposure to OCPs can also interfere with the functioning of the immune system [66], and γ-HCH, *cis-*CHL and 4,4′-DDT must be closely monitored due to their high potential risks associated with infertility and gynecological diseases. Aside from fertility studies, the association of OCPs to female reproductive-associated surgeries and technologies has also been investigated. Zhu, et al. [67] measured the follicular fluid in female patients who had undergone assisted reproductive treatments and observed high levels of OCPs (168 ng·g^−1^·lipid^−1^). Our results offer valuable information for encouraging future in-vitro, in-vivo, and epidemiological studies on associations between the body burden of γ-HCH and female infertility. 

Menstrual parameters, including average periods of menstrual cycle (≤26 and ≥30 days), average menstrual period days (>5 days), shortest menstrual period days (≤3 days), hormonal drug intake, and infertility in association with OCP residues in breast milk were investigated. Infertile women and those who had taken hormonal drugs had significantly high ORs of OCPs, while women with menstrual cycles of ≤3 days had significantly higher OCP ORs than those whose menstrual duration was greater. A low OR of endosulfan II was observed in women with irregular menstrual periods (≤26 and ≥30 days); however, there was no significant association observed. Currently, there is no epidemiological evidence associating OCPs with menstruation; however, there have been reports linking menstruation characteristics with other pollutants. In our previous studies [68,69], higher levels of dioxins and PBDEs were observed in women with irregular menstrual cycles as compared to those with regular cycles. Women whose cycles were longer than 33 days also had higher dioxin levels compared to those with less than 33 days [69]. We showed in this study that the effects of OCPs on the menstruation characteristics of women are similar to those for PBDEs and dioxins, as demonstrated in our previous studies. OCPs may have the potential to interfere with hormonal balance thus disrupting menstruation characteristics. 

The limitation of this work is that it was a part of an on-going follow-up study to examine the health effects after mother-infant pairs were exposed to organochlorines. It was not easy to show significance in the statistical analysis due to the small sample size (*n* = 68) and the fact that our participants might not have been willing to report that they had undergone gynecological surgery or had received treatment for infertility. Although several significances were found in the tests using a logistic regression and a PCA model, these statistical methods were possibly not the best choices for the examination of the associations of OCPs to health outcomes in the present study, which was not a case-control designed study. More detailed information is provided in the Supplemental Materials section (please check the limitation of small sample size section).

The OCP levels in the breast milk in this study are relatively low and within safe levels, suggesting a low risk to human health. Similarly, Chang [5] indicated that the risk for potential adverse health effects of OCPs in terms of EDI in Taiwan is 1% lower than the acceptable value set by the Food and Agriculture Organization of the World Health Organization (DDT: 20 µg·kg^−1^ of body weight), also revealing a low risk to Taiwanese health (within safe levels). The levels of breast milk OCP may reflect the body burden in women of child-bearing ages and their effects on nursing infants and adults (i.e., the positive association of breast cancer mortality rate to OCP levels in Taiwanese women, especially for those who were born before the year 1975 [53]), and this information is also important for the longitudinal biomonitoring of OCPs and their body burdens in humans. The breast milk OCP concentrations were within the safe levels; however, there was a potential risk of developing gynecological disorders upon exposure to these compounds. Although body burden of OCPs is a minor contributor to gynecological disorders, several reports indicated the risks of endometriosis [70]) and breast cancer [71,72], or the potential effects on ovarian function [73,74] increased after women were exposed to endocrine disrupting compounds (EDCs) or persistent organic pollutants (POPs) including OCPs. Therefore, it is still necessary to continuously monitor the breast milk OCPs with low levels. This study provides useful and valuable information about high levels of certain OCPs in breast milk, which possibly may induce reproductive alterations in female adults. In our future studies, we will continuously monitor the OCP levels in breast milk and create longitudinal and large-scale epidemiological studies for examining the associations of breast milk OCPs with reproduction in Taiwan. 

## 5. Conclusions

Even after the long-term ban of OCPs use in Taiwan, the OCP residues in human breast milk samples were detectable. The most dominant OCP species was ΣDDT, followed by ΣHCH. Our results were significantly lower as compared to those observed in some developing and developed countries. Sociodemographic characteristics especially annual family income and gravidity were found to be correlated to several OCPs, particularly ΣHCH. In addition to these two sociodemographic characteristics, dietary habits such as consumptions of cow milk and beef were also associated with ΣHCH. Moreover, other OCPs such as *cis-*CHL and γ-HCH were associated with menstruation characteristics, hormonal drug intake, infertility treatment, cow milk consumption, and beef consumption. Accordingly, dietary habit is an important factor influencing the levels of OCPs in breast milk and the associated risks for women. 

## Figures and Tables

**Table 1:** Descriptive statistics of demographic or sociodemographic characteristics in the pregnant women (*n* = 68).

Subject Characteristics	Range	Mean ± SD
Maternal age (years)	19–40	30.5 ± 4.30
Pre-pregnant BMI (kg·m^−2^)	15.4–34.9	22.5 ± 4.11
Duration of residence (years)	1–39	21.8 ± 11.8
Parity (number)	1–5	2.21 ± 0.939
	Frequency (person)	Percentage
Birth year		
≤1975 year	27	39.7
>1975 year	41	60.3
Gravidity		
primiparous	16	23.5
multiparous	52	77.5
Population		
Native-born Taiwanese	51	75.0
Native-born Aborigines	12	17.6
Nonnative-born Taiwanese ^a^	5	7.40
Education levels		
Pre-senior high school	6	8.8
Senior high school	27	39.7
Tertiary education	31	45.6
Graduate education	4	5.9
Annual Income (U.S. Dollars)		
Less than 10,000	10	14.7
Between 10,000 and 20,000	22	32.4
Between 20,000 and 34,000	25	36.8
More than 34,000	11	16.2
Duration of breastfeeding		
≤2 months	2	2.9
>2 months	66	97.1
Infertility medical treatment ^b^		
Yes	9	13.2
No	59	86.8
Gynecological surgery ^b^		
Yes	8	11.8
No	60	88.2

^a^ They are foreign spouses from Vietnam or China. ^b^ The subjects were medically treated for infertility or had undergone gynecological surgery.

**Table 2:** Descriptive statistics of dietary habit and menstruation characteristics in the pregnant women (*n* = 68).

	Frequency (Person)	Percentage
Dietary habit		
Milk consumption		
≤625 (mL·week^−1^)	51	75.0
>625 (mL·week^−1^)	17	25.0
Cheese consumption		
≤1 (piece·week^−1^)	60	88.2
>1 (piece·week^−1^)	8	11.8
Egg consumption		
≤5 (piece·week^−1^)	38	55.9
>5 (piece·week^−1^)	30	44.1
Beef consumption		
≤50 (gram·week^−1^)	55	80.9
>50 (gram·week^−1^)	13	19.1
Pork consumption		
≤250 (gram·week^−1^)	44	64.7
>250 (gram·week^−1^)	24	35.3
Chicken consumption		
≤125 (gram·week^−1^)	36	52.9
>125 (gram·week^−1^)	32	47.1
Menstruation characteristics		
Menarche		
Before 13 years old	42	61.8
After 13 years old	26	38.2
Average periods of menstrual cycles		
27 to 29 days	30	44.1
≤26 and ≥30 days	38	55.9
Average menstrual period days		
≤5 days	32	47.1
>5 days	36	52.9
Shortest menstrual period days		
≤3 days	20	29.4
>3 days	48	70.6

**Table 3:** Concentrations (ng·g^−1^·lipid^−1^) of OCP residues in breast milk (*n* = 68).

	Detection Rate (%)	Range	Median	GM ^a^ ± SD
Aldrin	93	<MDL ^c^ (0.0241)–2.32	0.157	0.168 ± 0.455
ΣHCH ^b^	100	0.110–2.97	0.509	0.539 ± 0.557
α-HCH	91	<MDL (0.0416)–1.64	0.127	0.133 ± 0.290
β-HCH	91	<MDL (0.0376)–1.33	0.115	0.120 ± 0.227
γ-HCH	88	<MDL (0.0320)–0.616	0.0967	0.0914 ± 0.107
δ-HCH	82	<MDL (0.0347)–0.472	0.103	0.0945 ± 0.119
ΣCHL ^a^	93	<MDL (0.0753)–1.90	0.134	0.161 ± 0.284
*cis-*Chlordane (*cis-*CHL)	78	<MDL (0.0346)–0.730	0.0804	0.0740 ± 0.124
*trans-*Chlordane (*trans-*CHL)	52	<MDL (0.0407)–1.90	0.0538	0.0564 ± 0.251
ΣDDT ^a^	100	0.720–51.4	10.9	9.81 ± 7.52
4,4-DDD	56	<MDL (0.0271)–8.38	0.229	0.161 ± 1.64
4,4-DDE	100	0.348–44.6	9.24	8.07 ± 6.53
4,4-DDT	85	<MDL (0.0536)–3.57	0.414	0.360 ± 0.798
Dieldrin	96	<MDL (0.0199)–2.87	0.165	0.170 ± 0.500
ΣEndosulfan ^a^	100	0.0155–5.43	0.301	0.290 ± 0.776
Endosulfan I	66	<MDL (0.0440)–2.20	0.0908	0.0890 ± 0.377
Endosulfan II	46	<MDL (0.0322)–2.51	0.0161	0.0497 ± 0.379
Endosulfan sulfate	85	<MDL (0.0155)–0.735	0.0658	0.0713 ± 0.169
ΣEndrin ^a^	99	<MDL (0.108)–4.61	0.248	0.381 ± 0.701
Endrin	93	<MDL (0.0449)–3.04	0.165	0.176 ± 0.435
Endrin aldehyde	69	<MDL (0.0247)–1.56	0.0633	0.0618 ± 0.261
Endrin ketone	52	<MDL (0.0387)–0.867	0.0465	0.0594 ± 0.180
ΣHeptachlor ^a^	100	0.0728–5.01	0.705	0.645 ± 0.995
Heptachlor	100	0.0412–3.48	0.372	0.376 ± 0.667
Heptachlor epoxide (isomer B)	94	<MDL (0.0316)–1.53	0.227	0.217 ± 0.374
Methoxychlor	47	<MDL (0.0245)–0.618	0.0123	0.0388 ± 0.145

^a^ GM: geometric mean. ^b^ ΣHCH is the sum of α, β, γ, and δ-HCH; ΣCHL is the sum of *cis-* and *trans-*CHL; ΣDDT is the sum of 4,4′-DDD, 4,4′-DDE, and 4,4′-DDT; Σendosulfan is the sum of endosulfanI, endosulfan II, and endosulfan sulfate; Σendrin is the sum of endrin, endrinaldehyde, and endrinketone; Σheptachlor is the sum of heptachlor and heptachlor epoxide. ^c^ MDL: method detection limit.

**Table 4:** Significant associations between milk OCPs and dietary habit and menstruation characteristics using a stepwise linear regression.

Independent	Dependent	Adjustment ^a^			
	Predictor	Adjusted R^2^	B	95% CI for B ^b^	*p* Value
Dietary habit					
Milk consumption	Log Endrin ketone	0.053	0.308	(90.3, 863)	0.016 *
Beef consumption		0.313			<0.001 ***
	Log Endosulfan sulfate		0.366	(12.8, 48.6)	0.001 **
	Log *cis-*CHL		0.333	(10.5, 62.9)	0.007 **
	Log γ-HCH		0.276	(−60.1, −7.72)	0.012 *
	Log Endrin ketone		0.284	(5.64, 42.5)	0.011 *
	Log Endrin		0.278	(−49.9, −5.27)	0.016 *
Menstruation characteristics					
Shortest menstrual period days	Log *trans-*CHL	0.120	0.279	(0.109, 1.40)	0.023 *

^a^ Adjusted by pre-pregnant BMI, age, population, annual income, birth year and parity. ^b^ 95% confidence intervals. * *p* < 0.05, ** *p* < 0.01,*** *p* < 0.001.

**Table 5:** Sociodemographic characteristics, dietary habits and menstruation characteristics of mothers in association with OCP residues in breast milk as determined using the logistic regression model.

	Odds Ratio ^a^	95% Confidence Intervals	*p*-Value
Population (native-born Aborigines) ^b^			
Log Endosulfan sulfate	10.8 ^c^	1.03–113	0.047 *
Annual family income (≤$20,000 U.S. dollar) ^b^			
Log α-HCH	4.20 ^d^	1.08–16.2	0.037 *
Log ΣHCH	10.7 ^d^	1.27–90.1	0.029 *
Gravidity (primiparous) ^b^			
Log ΣHCH	27.3 ^e^	1.63–457	0.021 *
Cow milk consumption (>625 mL·week^−1^) ^b^			
Log β-HCH	7.35	1.45–37.3	0.016 *
Log ΣCHL	6.65	1.10–40.1	0.039 *
Log Endosulfan I	3.03	1.04–8.83	0.043 *
Log Endosulfan II	4.53	1.56–13.1	0.005 **
Log ΣEndosulfan	6.67	1.53–28.9	0.011 *
Log Endrin	6.73	1.32–34.4	0.022 *
Log Endrin ketone	3.72	1.18–11.7	0.025 *
Log ΣEndrin	13.3	2.05–86.1	0.007 **
Log ΣHeptachlor	9.11	1.72–48.4	0.010 *
Beef consumption (>50 g·week^−1^) ^b^			
Log γ-HCH	0.128	0.017–0.946	0.044 *
Log *trans-*CHL	5.05	1.25–20.4	0.023 *
Log ΣCHL	10.5	1.36–81.2	0.024 *
Log Endosulfan II	4.18	1.27–13.8	0.019 *
Log Endosulfan sulfate	7.16	1.36–37.7	0.020 *
Log ΣEndosulfan	6.88	1.24–38.0	0.027 *
Log Methoxychlor	4.01	1.13–14.2	0.032 *
Average menstrual period days (>5 days) ^b^			
Log *trans-*CHL	4.73	1.12–20.1	0.035 *
Log Endrin ketone	7.06	1.58–31.6	0.011 *
Shortest menstrual period days (≤3 days) ^b^			
Log *trans-*CHL	14.9	1.53–145	0.020 *
Log ΣCHL	14.5	1.07–197	0.044 *
Have taken hormonal drugs ^b^			
Log ΣEndosulfan	18.6	1.22–283	0.035 *
Log Heptachlor epoxide (isomer B)	16.6	1.72–160	0.015 *
Log ΣHeptachlor	21.6	1.07–437	0.045 *
Infertility ^b^			
Log γ-HCH	25.6	1.26–519	0.035 *

^a^ Adjusted for pre-pregnant BMI, age, population, annual income, birth year, and parity. ^b^ The reference groups are native-born and nonnative-born Taiwanese, lower annual family income (<$20,000 U.S. dollars), multiparous mothers, women having lower consumption of cow milk (<625 mL·week^−1^), mothers having lower consumption of beef (<50 g·week^−1^), women with normal period of averaged menstrual period, women with normal period of the shortest menstrual period, mothers without taking hormonal drugs, and women without experience with infertility treatment. ^c^ Adjusted by pre-pregnant BMI, age, annual income, birth year and parity. ^d^ Adjusted by pre-pregnant BMI, age, population, birth year and parity. ^e^ Adjusted by pre-pregnant BMI, age, population, annual income, and birth year. * *p* < 0.05, ** *p* < 0.01.

**Table 6:** OCP levels in breast milk in different countries.

Country	Calendar Period	Measured Compound	Pesticide Level	Reference
Taiwan	2000–2001	Aldrin Dieldrin Endrin β-HCH γ-HCH α-CHL Heptachlor Heptachlor epoxide *p,p*′-DDT *p,p*′-DDE	<LOD <LOD <LOD 1.2 ng·g^−1^·lipid^−1^ 0.8 ng·g^−1^·lipid^−1^ 7.4 ng·g^−1^·lipid^−1^ 2.3 ng· g^−1^·lipid^−1^ 4.0 ng·g^−1^·lipid^−1^ 19 ng·g^−1^·lipid^−1^ 228 ng·g^−1^·lipid^−1^	Chao, et al. [18]
Taiwan	2013–2016	Aldrin ΣHCH ^a^ α-HCH β-HCH γ-HCH δ-HCH ΣCHL ^a^ *cis-*Chlordane (*cis-*CHL) *trans-*Chlordane (*trans-*CHL) ΣDDT ^a^ 4,4-DDD 4,4-DDE 4,4-DDT Dieldrin ΣEndosulfan ^a^ Endosulfan I Endosulfan II Endosulfan sulfate ΣEndrin ^a^ Endrin Endrin aldehyde Endrin ketone ΣHeptachlor ^a^ Heptachlor Heptachlor epoxide (isomer B) Methoxychlor	<MDL (0.0241)–2.32 0.110–2.97 <MDL (0.0416)–1.64 <MDL (0.0376)–1.33 <MDL (0.0320)–0.616 <MDL (0.0347)–0.472 <MDL–1.90 <MDL (0.0346)–0.730 <MDL (0.0407)–1.90 0.720–51.4 <MDL (0.0271)–8.38 0.348–44.6 <MDL (0.0536)–3.57 <MDL (0.0199)–2.87 0.0155–5.43 <MDL (0.0440)–2.20 <MDL (0.0322)–2.51 <MDL (0.0155)–0.735 <MDL–4.61 <MDL (0.0449)–3.04 <MDL (0.0247)–1.56 <MDL (0.0387)–0.867 0.0728–5.01 0.0412–3.48 <MDL (0.0316)–1.53 <MDL (0.0245)–0.618	This study
Thailand	1998	Heptachlor Heptachlor epoxide γ-HCH *p,p*′-DDT *p,p*′-DDE *p,p*′-DDD	4.3 ng·mL^−1^ 4.4 ng·mL^−1^ 3.6 ng·mL^−1^ 69.4 ng·mL^−1^ 169.4 ng·mL^−1^ 6.8 ng·mL^−1^	Stuetz, et al. [23]
China	1999–2000	*p,p*′-DDT *p,p*′-DDE β-HCH	0.70 μg·g^−1^·fat^−1^ 2.85 μg·g^−1^·fat^−1^ 1.11 μg·g^−1^·fat^−1^	Wong, et al. [24]
China	2003–2005	α-HCH γ-HCH β-HCH *p,p*′-DDE	76.16 ng·g^−1^·lipid^−1^ 16.67 ng·g^−1^·lipid^−1^ 214.33 ng·g^−1^·lipid^−1^ 1528.20 ng·g^−1^·lipid^−1^	Zhao, et al. [25]
China	2006, 2008, 2010	α-HCH β-HCH γ-HCH δ-HCH HCB 2,4′-DDE 4,4′-DDE 2,4′-DDD 4,4′-DDD	<LOD 67.1 ng·g^−1^·lipid^−1^ <LOD >LOD 25.5 ng·g^−1^·lipid^−1^ <LOD <LOD <LOD 10.5 ng·g^−1^·lipid^−1^	Zhou, et al. [26]
Vietnam	2000–2001	*p,p*′-DDT *p,p*′-DDE *p,p*′-DDD β-HCH	34 –6900 ng·g^−1^·lipid·wt^−1^ 420–6300 ng·g^−1^·lipid·wt^−1^ 3–50 ng·g^−1^·lipid·wt^−1^ 11–160 ng·g^−1^·lipid·wt^−1^	Minh, et al. [27]
Korea	2011	*p,p*′-DDT *p,p*′-DDE *p,p*′-DDD β-HCH Heptachlor epoxide	91.7 ng·g^−1^·lipid·wt^−1^ 0.94 ng·g^−1^·lipid·wt^−1^ 6.51 ng·g^−1^·lipid·wt^−1^ 20.5 ng·g^−1^·lipid·wt^−1^ 2.22 ng·g^−1^·lipid·wt^−1^	Lee, et al. [29]
USA, Mexico and Russia	1999, 2002, 2007, 2009, 2011	HCB β-HCH *p,p*′-DDT *p,p*′-DDE	0.80–3.00 ng·g^−1^·lipid·wt^−1^ 0.51–2.57 ng·g^−1^ ·lipid·wt^−1^ 0.42–1.41 ng·g^−1^·lipid·wt^−1^ 0.56–1.40 ng·g^−1^·lipid·wt^−1^	Coakley, et al. [32]
USA	2004	*p,p*′-DDT *p,p*′-DDE *p,p*′-DDD α-HCH β-HCH γ-HCH δ-HCH	<0.6 ng·g^−1^·lipid·wt^−1^ 35.3 ng·g^−1^·lipid·wt^−1^ 2.7 ng·g^−1^·lipid·wt^−1^ 1.4 ng·g^−1^ lipid·wt^−1^ 4.4 ng·g^−1^·lipid·wt^−1^ 5.1 ng·g^−1^·lipid·wt^−1^ <1.6 ng·g^−1^·lipid·wt^−1^	Johnson-Restrepo, et al. [33]
Russia	1997–2009	HCB α-HCH γ-HCH *p,p*′-DDT *p,p*′-DDE *p,p*′-DDD	29 ng·g^−1^·lipid^−1^ 3.1 ng·g^−1^·lipid^−1^ 0.56 ng·g^−1^·lipid^−1^ 32 ng·g^−1^·lipid^−1^ 491 ng·g^−1^·lipid^−1^ 1.9 ng·g^−1^·lipid^−1^	Mamontova, et al. [34]
Colombia	Unspecified	4,4′ DDE 4,4′ DDE	126 ng·g·lipid·wt 203 ng·g·lipid·wt	Rojas-Squella, et al. [35]
Norway	2002–2006	*p,p*′-DDE HCB β-HCH Oxychlordane	41 ng·g·lipid·wt 11 ng·g·lipid·wt 4.7 ng·g·lipid·wt 2.8 ng·g·lipid·wt	Polder, et al. [36]
Vietnam, China, and Japan	2007–2008	*p,p*′-DDT *p,p*′-DDE *p,p*′-DDD *o,p*′-DDT Oxychlordane β-HCH HCB	5.8 ng·g^−1^·lipid^−1^ 160 ng·g^−1^·lipid^−1^ 1.4 ng·g^−1^·lipid^−1^ 0.84 ng·g^−1^·lipid^−1^ 3.7 ng·g^−1^·lipid^−1^ 140 ng·g^−1^·lipid^−1^ 13 ng·g^−1^·lipid^−1^	Haraguchi, et al. [28]

MDL: method detection limit. ^a^ ΣHCH is the sum of α, β, γ, and δ-HCH; ΣCHL is the sum of *cis-* and *trans-*CHL; ΣDDT is the sum of 4,4′-DDD, 4,4′-DDE, and 4,4′-DDT; Σendosulfan is the sum of endosulfan I, endosulfan II, and endosulfan sulfate; Σendrin is the sum of endrin, endrinaldehyde, and endrin ketone; Σheptachlor is the sum of heptachlor and heptachlor epoxide.

## Supplementary Materials

The following are available online at http://www.mdpi.com/1660-4601/15/5/931/s1, Table S1: Breast milk OCP levels (ng/g lipid) of Taiwanese women who were born before, in, and after 1975, Table S2: Odds ratios of OCP residues in breast milk and their correlation to native-born Aborigines (*n* = 12) in comparison to native-born and nonnative-born Taiwanese (*n* = 56) as determined by the logistic regression model, Table S3: Odds ratios of OCP residues in breast milk and their correlation to women who were born before and during 1975 (*n* = 27) in comparison to women born after 1975 (*n* = 41) as determined by the logistic regression model, Table S4: Odds ratios of OCP residues in breast milk and their correlation to older mothers (>31 years old) in comparison to younger mothers (≤31 years old) as determined by the logistic regression model, Table S5: Odds ratios of OCP residues in breast milk and their correlation to mothers who have higher pre-pregnant BMI values (>21.7 kg m^−2^) in comparison to those having lower pre-pregnant BMI values (≤21.7 kg m^−2^) as determined by the logistic regression model, Table S6: Odds ratio of OCP residues in breast milk and their correlation to mothers having low annual family income (≤$20,000 US dollar) in comparison to mothers having high annual family income (>$20,000 US dollar) as determined by the logistic regression model, Table S7: Odds ratio of OCP residues in breast milk and their correlation to primiparous mothers in comparison to mothers who are multiparous as determined by the logistic regression model, Table S8: Odds ratio of OCP residues in breast milk and their correlation to mothers who have attained low education levels (pre-senior and senior high school) in comparison to those who with high education background (tertiary education and graduate education) as determined by the logistic regression model, Table S9: Associations of poultry (egg) and dairy products (milk and cheese) consumption to breastmilk OCPs as determined by Mann-Whitney *U* Test, Table S10: Meat (beef, pork, and chicken) consumption in correlation to breast milk OCPs as determined by Mann-Whitney *U* Test, Table S11: Odds ratios of OCP residues in breast milk and their correlation to cow milk consumption as determined by logistic regression models, Table S12: Odds ratios of OCP residues in breast milk and their correlation to beef consumption as determined by logistic regression models, Table S13: Odds ratios of OCP residues in breast milk and their correlation to pork consumption as determined by logistic regression models, Table S14: Odds ratios of OCP residues in breast milk and their correlation to chicken consumption as determined by logistic regression models, Table S15: Odds ratios of OCP residues in breast milk and their associations to mothers who menarche before 13 years old in comparison to mothers who menarche after 13 years old as determined by logistic regression models, Table S16: Odds ratio of average periods of menstrual cycles of 27 to 29 days as compared to menstrual cycles of ≤26 and ≥30 days as determined by logistic regression models, Table S17: Odds ratio of breast milk OCP residues and their associations to women with average menstrual period days of >5 days as determined by logistic regression models, Table S18: Odds ratio of breast milk OCP residues and their associations to women with the shortest menstrual period days of ≤3 days as determined by logistic regression models, Table S19: Odds ratio of breast milk OCP residues and their associations to women who have taken contraceptives as determined by logistic regression models, Table S20: Odds ratio of breast milk OCP residues and their associations to women who have or have not taken hormonal drugs as determined by logistic regression models, Table S21: Odds ratios of OCP residues in breast milk from mothers who received infertility medical treatment in comparison to normal mothers as determined by logistic regression models, Table S22: Odds ratios of breast milk OCP residues in the participants having undergone gynecological surgery compared with those in normal women as determined by logistic regression models, Table S23: Sociodemographic characteristics, dietary habits and menstruation characteristics of mothers in association with OCP residues in breast milk as determined using the logistic regression model, Supplemental text: Limitation of small sample size.

## References

[1] Coker E., Chevrier J., Rauch S., Bradman A., Obida M., Crause M., Bornman R., Eskenazi B. (2018). Association between prenatal exposure to multiple insecticides and child body weight and body composition in the VHEMBE South African birth cohort. Environ. Int..

[2] Ntow W.J., Tagoe L.M., Drechsel P., Kelderman P., Gijzen H.J., Nyarko E. (2008). Accumulation of persistent organochlorine contaminants in milk and serum of farmers from Ghana. Environ. Res..

[3] Smith A., Gangolli S. (2002). Organochlorine chemicals in seafood: Occurrence and health concerns. Food Chem. Toxicol..

[4] Aktar W., Sengupta D., Chowdhury A. (2009). Impact of pesticides use in agriculture: Their benefits and hazards. Interdiscip. Toxicol..

[5] Chang G.-R. (2018). Persistent organochlorine pesticides in aquatic environments and fishes in Taiwan and their risk assessment. Environ. Sci. Pollut. Res..

[6] Okoya A.A., Ogunfowokan A.O., Asubiojo O.I., Torto N. (2013). Organochlorine pesticide residues in sediments and waters from cocoa producing areas of Ondo State, Southwestern Nigeria. ISRN Soil Sci..

[7] Tsai W.-T. (2010). Current status and regulatory aspects of pesticides considered to be persistent organic pollutants (POPs) in Taiwan. Int. J. Environ. Res. Public Health.

[8] Meeker J.D., Altshul L., Hauser R. (2007). Serum PCBs, *p*,*p*′-DDE and HCB predict thyroid hormone levels in men. Environ. Res..

[9] Trapp M., Baukloh V., Bohnet H.-G., Heeschen W. (1984). Pollutants in human follicular fluid. Fertil. Steril. (United States).

[10] Murono E.P., Derk R.C., Akgul Y. (2006). In vivo exposure of young adult male rats to methoxychlor reduces serum testosterone levels and ex vivo Leydig cell testosterone formation and cholesterol side-chain cleavage activity. Reprod. Toxicol..

[11] Lindenau A., Fischer B., Seiler P., Beier H.M. (1994). Effects of persistent chlorinated hydrocarbons on reproductive tissues in female rabbits. Hum. Reprod..

[12] Kadhel P., Monnier P., Boucoiran I., Chaillet N., Fraser W.D. (2012). Organochlorine Pollutants and Female Fertility A Systematic Review Focusing on In Vitro Fertilization Studies. Reprod. Sci..

[13] Weiss J.M., Bauer O., Blüthgen A., Ludwig A.K., Vollersen E., Kaisi M., Al-Hasani S., Diedrich K., Ludwig M. (2006). Distribution of persistent organochlorine contaminants in infertile patients from Tanzania and Germany. J. Assist. Reprod. Genet..

[14] Axmon A., Thulstrup A.-M., Rignell-Hydbom A., Pedersen H., Zvyezday V., Ludwicki J., Jönsson B., Toft G., Bonde J.-P., Hagmar L. (2006). Time to pregnancy as a function of male and female serum concentrations of 2, 2′ 4, 4′ 5, 5′-hexachlorobiphenyl (CB-153) and 1,1-dichloro-2,2-bis (*p*-chlorophenyl)-ethylene (*p*,*p*′-DDE). Hum. Reprod..

[15] Bastos A.M.X., Souza Mdo C., Almeida Filho G.L., Krauss T.M., Pavesi T., Silva L.E. (2013). Organochlorine compound levels in fertile and infertile women from Rio de Janeiro, Brazil. Arq. Bras. Endocrinol. Metab..

[16] Ribas-Fitó N., Cardo E., Sala M., De Muga M.E., Mazón C., Verdu A., Kogevinas M., Grimalt J.O., Sunyer J. (2003). Breastfeeding, exposure to organochlorine compounds, and neurodevelopment in infants. Pediatrics.

[17] Longnecker M.P., Klebanoff M.A., Zhou H., Brock J.W. (2001). Association between maternal serum concentration of the DDT metabolite DDE and preterm and small-for-gestational-age babies at birth. Lancet.

[18] Chao H.-R., Wang S.-L., Lin T.-C., Chung X.-H. (2006). Levels of organochlorine pesticides in human milk from central Taiwan. Chemosphere.

[19] Li G., Wu L., Flanagan M. (1983). The survey of ogranochlorine pesticide residues in breast milk from Taiwan. Plant Prot. Bull..

[20] Jaga K., Dharmani C. (2003). Global surveillance of DDT and DDE levels in human tissues. Int. J. Occup. Med. Environ. Health.

[21] Kang J.-H., Park H., Chang Y.-S., Choi J.-W. (2008). Distribution of organochlorine pesticides (OCPs) and polychlorinated biphenyls (PCBs) in human serum from urban areas in Korea. Chemosphere.

[22] Quintana P.J., Delfino R.J., Korrick S., Ziogas A., Kutz F.W., Jones E.L., Laden F., Garshick E. (2004). Adipose tissue levels of organochlorine pesticides and polychlorinated biphenyls and risk of non-Hodgkin’s lymphoma. Environ. Health Perspect..

[23] Stuetz W., Prapamontol T., Erhardt J., Classen H. (2001). Organochlorine pesticide residues in human milk of a Hmong hill tribe living in Northern Thailand. Sci. Total Environ..

[24] Wong C., Leung K., Poon B., Lan C., Wong M. (2002). Organochlorine hydrocarbons in human breast milk collected in Hong Kong and Guangzhou. Arch. Environ. Contam. Toxicol..

[25] Zhao G., Xu Y., Li W., Han G., Ling B. (2007). PCBs and OCPs in human milk and selected foods from Luqiao and Pingqiao in Zhejiang, China. Sci. Total Environ..

[26] Zhou J., Zeng X., Zheng K., Zhu X., Ma L., Xu Q., Zhang X., Yu Y., Sheng G., Fu J. (2012). Musks and organochlorine pesticides in breast milk from Shanghai, China: Levels, temporal trends and exposure assessment. Ecotoxicol. Environ. Saf..

[27] Minh N.H., Someya M., Minh T.B., Kunisue T., Iwata H., Watanabe M., Tanabe S., Viet P.H., Tuyen B.C. (2004). Persistent organochlorine residues in human breast milk from Hanoi and Hochiminh City, Vietnam: Contamination, accumulation kinetics and risk assessment for infants. Environ. Pollut..

[28] Haraguchi K., Koizumi A., Inoue K., Harada K.H., Hitomi T., Minata M., Tanabe M., Kato Y., Nishimura E., Yamamoto Y. (2009). Levels and regional trends of persistent organochlorines and polybrominated diphenyl ethers in Asian breast milk demonstrate POPs signatures unique to individual countries. Environ. Int..

[29] Lee S., Kim S., Lee H.K., Lee I.S., Park J., Kim H.J., Lee J.J., Choi G., Choi S., Kim S. (2013). Contamination of polychlorinated biphenyls and organochlorine pesticides in breast milk in Korea: Time-course variation, influencing factors, and exposure assessment. Chemosphere.

[30] Sharma A., Gill J., Bedi J., Pooni P. (2014). Monitoring of pesticide residues in human breast milk from punjab, india and its correlation with health associated parameters. Bull. Environ. Contam. Toxicol..

[31] Hajjar M.J., Al-Salam A. (2016). Organochlorine pesticide residues in human milk and estimated daily intake (EDI) for the infants from eastern region of Saudi Arabia. Chemosphere.

[32] Coakley J., Mueller J.F., Harden F., Toms L.-M., Douwes J. (2012). Partitioning of persistent organic pollutants (POPs) between human serum and breast milk: A literature review. Chemosphere.

[33] Johnson-Restrepo B., Addink R., Wong C., Arcaro K., Kannan K. (2007). Polybrominated diphenyl ethers and organochlorine pesticides in human breast milk from Massachusetts, USA. J. Environ. Monit..

[34] Mamontova E.A., Tarasova E.N., Mamontov A.A. (2017). PCBs and OCPs in human milk in Eastern Siberia, Russia: Levels, temporal trends and infant exposure assessment. Chemosphere.

[35] Rojas-Squella X., Santos L., Baumann W., Landaeta D., Jaimes A., Correa J.C., Sarmiento O.L., Ramos-Bonilla J.P. (2013). Presence of organochlorine pesticides in breast milk samples from Colombian women. Chemosphere.

[36] Polder A., Skåre J.U., Skjerve E., Løken K., Eggesbø M. (2009). Levels of chlorinated pesticides and polychlorinated biphenyls in Norwegian breast milk (2002–2006), and factors that may predict the level of contamination. Sci. Total Environ..

[37] LaKind J.S., Berlin C.M., Naiman D.Q. (2001). Infant exposure to chemicals in breast milk in the United States: What we need to learn from a breast milk monitoring program. Environ. Health Perspect..

[38] Ritter R., Scheringer M., MacLeod M., Schenker U., Hungerbühler K. (2009). A multi-individual pharmacokinetic model framework for interpreting time trends of persistent chemicals in human populations: Application to a postban situation. Environ. Health Perspect..

[39] Zietz B.P., Hoopmann M., Funcke M., Huppmann R., Suchenwirth R., Gierden E. (2008). Long-term biomonitoring of polychlorinated biphenyls and organochlorine pesticides in human milk from mothers living in northern Germany. Int. J. Hyg. Environ. Health.

[40] Achour A., Derouiche A., Barhoumi B., Kort B., Cherif D., Bouabdallah S., Sakly M., Rhouma K.B., Touil S., Driss M.R. (2017). Organochlorine pesticides and polychlorinated biphenyls in human adipose tissue from northern Tunisia: Current extent of contamination and contributions of socio-demographic characteristics and dietary habits. Environ. Res..

[41] Sharma H.R., Kaushik A., Kaushik C.P. (2007). Pesticide residues in bovine milk from a predominantly agricultural state of Haryana, India. Environ. Monit. Assess..

[42] Pan J., Gai N., Tang H., Chen S., Chen D., Lu G., Yang Y. (2014). Organochlorine pesticides and polychlorinated biphenyls in grass, yak muscle, liver, and milk in Ruoergai high altitude prairie, the eastern edge of Qinghai-Tibet Plateau. Sci. Total Environ..

[43] Li J., Li N., Ma M., Giesy J.P., Wang Z. (2008). In vitro profiling of the endocrine disrupting potency of organochlorine pesticides. Toxicol. Lett..

[44] Bempah C.K., Donkor A.K. (2011). Pesticide residues in fruits at the market level in Accra Metropolis, Ghana, a preliminary study. Environ. Monit. Assess..

[45] Khan F.H., Ganesan P., Kumar S. (2010). Y Chromosome microdeletion and altered sperm quality in human males with high concentration of seminal hexachlorocyclohexane (HCH). Chemosphere.

[46] Pant N., Kumar R., Mathur N., Srivastava S., Saxena D., Gujrati V.R. (2007). Chlorinated pesticide concentration in semen of fertile and infertile men and correlation with sperm quality. Environ. Toxicol. Pharmacol..

[47] Pant N., Shukla M., Upadhyay A., Chaturvedi P., Saxena D., Gupta Y. (2014). Association between environmental exposure to p, p′-DDE and lindane and semen quality. Environ. Sci. Pollut. Res..

[48] Upson K., De Roos A.J., Thompson M.L., Sathyanarayana S., Scholes D., Barr D.B., Holt V.L. (2013). Organochlorine pesticides and risk of endometriosis: Findings from a population-based case-control study. Environ. Health Perspect. (Online).

[49] Chand S., Mustafa M., Banerjee B., Guleria K. (2014). CYP17A1 gene polymorphisms and environmental exposure to organochlorine pesticides contribute to the risk of small for gestational age. Eur. J. Obstet. Gynecol. Reprod. Biol..

[50] Elserougy S., Beshir S., Saad-Hussein A., AbouArab A. (2013). Organochlorine pesticide residues in biological compartments of healthy mothers. Toxicol. Ind. Health.

[51] Sharma E., Mustafa M., Pathak R., Guleria K., Ahmed R.S., Vaid N., Banerjee B. (2012). A case control study of gene environmental interaction in fetal growth restriction with special reference to organochlorine pesticides. Eur. J. Obstet. Gynecol. Reprod. Biol..

[52] Xu X., Dailey A.B., Talbott E.O., Ilacqua V.A., Kearney G., Asal N.R. (2010). Associations of serum concentrations of organochlorine pesticides with breast cancer and prostate cancer in US adults. Environ. Health Perspect..

[53] Ho M.-L., Hsiao Y.-H., Su S.-Y., Chou M.-C., Liaw Y.-P. (2015). Mortality of breast cancer in Taiwan, 1971–2010: Temporal changes and an age–period–cohort analysis. J. Obstet. Gynaecol..

[54] Airaksinen R., Rantakokko P., Eriksson J.G., Blomstedt P., Kajantie E., Kiviranta H. (2011). Association between type 2 diabetes and exposure to persistent organic pollutants. Diabetes Care.

[55] Hong S., Hwang J., Kim J.Y., Shin K.S., Kang S.J. (2015). Heptachlor induced nigral dopaminergic neuronal loss and Parkinsonism-like movement deficits in mice. Exp. Mol. Med..

[56] Steenland K., Mora A., Barr D., Juncos J., Roman N., Wesseling C. (2014). Organochlorine chemicals and neurodegeneration among elderly subjects in Costa Rica. Environ. Res..

[57] Torres-Sánchez L., Rothenberg S.J., Schnaas L., Cebrián M.E., Osorio E., del Carmen Hernández M., García-Hernández R.M., del Rio-Garcia C., Wolff M.S., López-Carrillo L. (2007). In utero p, p′-DDE exposure and infant neurodevelopment: A perinatal cohort in Mexico. Environ. Health Perspect..

[58] Dewan P., Jain V., Gupta P., Banerjee B.D. (2013). Organochlorine pesticide residues in maternal blood, cord blood, placenta, and breastmilk and their relation to birth size. Chemosphere.

[59] Li C., Cheng Y., Tang Q., Lin S., Li Y., Hu X., Nian J., Gu H., Lu Y., Tang H. (2014). The association between prenatal exposure to organochlorine pesticides and thyroid hormone levels in newborns in Yancheng, China. Environ. Res..

[60] Sunyer J., Torrent M., Garcia-Esteban R., Ribas-Fitó N., Carrizo D., Romieu I., Antó J., Grimalt J. (2006). Early exposure to dichlorodiphenyldichloroethylene, breastfeeding and asthma at age six. Clin. Exp. Allergy.

[61] Dewailly E., Ayotte P., Bruneau S., Gingras S., Belles-Isles M., Roy R. (2000). Susceptibility to infections and immune status in Inuit infants exposed to organochlorines. Environ. Health Perspect..

[62] Cohn B.A., Wolff M.S., Cirillo P.M., Sholtz R.I. (2007). DDT and breast cancer in young women: New data on the significance of age at exposure. Environ. Health Perspect..

[63] Greenlee A.R., Arbuckle T.E., Chyou P.-H. (2003). Risk factors for female infertility in an agricultural region. Epidemiology.

[64] Mostafalou S., Abdollahi M. (2017). Pesticides: An update of human exposure and toxicity. Arch. Toxicol..

[65] Thomas A., Toms L.-M.L., Harden F.A., Hobson P., White N.M., Mengersen K.L., Mueller J.F. (2017). Concentrations of organochlorine pesticides in pooled human serum by age and gender. Environ. Res..

[66] Lebel G., Dodin S., Ayotte P., Marcoux S., Ferron L.A., Dewailly E. (1998). Organochlorine exposure and the risk of endometriosis. Fertil. Steril..

[67] Zhu Y., Huang B., Li Q.X., Wang J. (2015). Organochlorine pesticides in follicular fluid of women undergoing assisted reproductive technologies from central China. Environ. Pollut..

[68] Chao H.R., Shy C.G., Wang S.L., Chen S.C., Koh T.W., Chen F.A., Chang-Chien G.P., Tsou T.C. (2010). Impact of non-occupational exposure to polybrominated diphenyl ethers on menstruation characteristics of reproductive-age females. Environ. Int..

[69] Chao H.R., Wang S.L., Lin L.Y., Lee W.J., Papke O. (2007). Placental transfer of polychlorinated dibenzo-p-dioxins, dibenzofurans, and biphenyls in Taiwanese mothers in relation to menstrual cycle characteristics. Food Chem. Toxicol. Int. J. Publ. Br. Ind. Biol. Res. Assoc..

[70] Porpora M.G., Medda E., Abballe A., Bolli S., De Angelis I., di Domenico A., Ferro A., Ingelido A.M., Maggi A., Panici P.B. (2009). Endometriosis and organochlorinated environmental pollutants: A case-control study on Italian women of reproductive age. Environ. Health Perspect..

[71] Ingber S.Z., Buser M.C., Pohl H.R., Abadin H.G., Murray H.E., Scinicariello F. (2013). DDT/DDE and breast cancer: A meta-analysis. Regul. Toxicol. Pharmacol. RTP.

[72] Wielsøe M., Kern P., Bonefeld-Jørgensen E.C. (2017). Serum levels of environmental pollutants is a risk factor for breast cancer in Inuit: A case control study. Environ. Health.

[73] Shah H.K., Bhat M.A., Sharma T., Banerjee B.D., Guleria K. (2018). Delineating Potential Transcriptomic Association with Organochlorine Pesticides in the Etiology of Epithelial Ovarian Cancer. Open Biochem. J..

[74] Windham G.C., Lee D., Mitchell P., Anderson M., Petreas M., Lasley B. (2005). Exposure to Organochlorine Compounds and Effects on Ovarian Function. Epidemiology.

