# Supplementary material for: Association between Organochlorine Pesticide Levels in Breast Milk and Their Effects on Female Reproduction in a Taiwanese Population
Source: Int J Environ Res Public Health. 2018 May 7;15(5):931. doi: 10.3390/ijerph15050931 (PMC5981970; doi:10.3390/ijerph15050931)
Supplement: Supplementary file 1 [file ijerph-15-00931-s001.pdf]

International Journal of Environmental Research and Public Health
Supplementary Materials

Association between organochlorine pesticide levels in breast milk and their effects on female reproduction in a Taiwanese population

Men-Wen Chen[1], Harvey M. Santos[2], Danielle E. Que[3], Yan-You Gou [1,+], Lemmuel L. Tayo[2], Yi-Chyun Hsu[4], Young-Bin Chen[5], Fu-An Chen[6,+,] How-Ran Chao [1,7,+], and Kuo-Lin Huang[1,*,+]

[1] Emerging Compounds Research Center, Department of Environmental Science and Engineering, National Pingtung University of Science and Technology, Neipu, Pingtung County 912, Taiwan
[2] School of Chemical Engineering, Chemistry, Biological Engineering and Material Science and Engineering, Mapúa Institute of Technology, Muralla St., Intramuros, Manila 1002, Philippines
[3] Department of Environmental Engineering, National Cheng Kung University, Tainan City 701, Taiwan
[4] Department of Environmental Engineering, Kun Shan University, Tainan City 710, Taiwan
[5] Department of Biological Science and Technology, National Pingtung University of Science and Technology, Neipu, Pingtung County 912, Taiwan
[6] Department of Pharmacy & Graduate Institute of Pharmaceutical Technology, Tajen University, Pingtung 90741, Taiwan
[7] Institute of Food Safety Management, National Pingtung University of Science and Technology, Neipu, Pingtung County 912, Taiwan

+ These authors equally contributed.
* **Correspondence**: Kuo-Lin Huang, huangkL@mail.npust.edu.tw; Tel.: +886-87703202 ext. 7092

26 pages

23 tables
1 text

Index

Table S1
Breast milk OCP levels (ng/g lipid) of Taiwanese women who were born before, in, and after 1975
Table S2
Odds ratios of OCP residues in breast milk and their correlation to native-born Aborigines (n = 12) in comparison to native-born and nonnative-born Taiwanese (n = 56) as determined by the logistic regression model
Table S3
Odds ratios of OCP residues in breast milk and their correlation to women who were born before and during 1975 (n = 27) in comparison to women born after 1975 (n = 41) as determined by the logistic regression model
Table S4
Odds ratios of OCP residues in breast milk and their correlation to older mothers (>31 years old) in comparison to younger mothers (≤31 years old) as determined by the logistic regression model
Table S5
Odds ratios of OCP residues in breast milk and their correlation to mothers who have higher pre-pregnant BMI values (>21.7 kg m^{-2}) in comparison to those having lower pre-pregnant BMI values (≤21.7 kg m^{-2}) as determined by the logistic regression model
Table S6
Odds ratio of OCP residues in breast milk and their correlation to mothers having low annual family income (≤$20,000 US dollar) in comparison to mothers having high annual family income (>$20,000 US dollar) as determined by the logistic regression model
Table S7
Odds ratio of OCP residues in breast milk and their correlation to primiparous mothers in comparison to mothers who are multiparous as determined by the logistic regression model
Table S8
Odds ratio of OCP residues in breast milk and their correlation to mothers who have attained low education levels (pre-senior and senior high school) in comparison to those who with high education background (tertiary education and graduate education) as determined by the logistic regression model
Table S9
Associations of poultry (egg) and dairy products (milk and cheese) consumption to breastmilk OCPs as determined by Mann-Whitney *U* Test
Table S10
Meat (beef, pork, and chicken) consumption in correlation to breast milk OCPs as determined by Mann-Whitney *U* Test
Table S11

Odds ratios of OCP residues in breast milk and their correlation to cow milk consumption as determined by logistic regression models
Table S12
Odds ratios of OCP residues in breast milk and their correlation to beef consumption as determined by logistic regression models
Table S13
Odds ratios of OCP residues in breast milk and their correlation to pork consumption as determined by logistic regression models
Table S14
Odds ratios of OCP residues in breast milk and their correlation to chicken consumption as determined by logistic regression models
Table S15
Odds ratios of OCP residues in breast milk and their associations to mothers who menarche before 13 years old in comparison to mothers who menarche after 13 years old as determined by logistic regression models
Table S16
Odds ratio of average periods of menstrual cycles of 27 to 29 days as compared to menstrual cycles of ≤26 and ≥30 days as determined by logistic regression models
Table S17
Odds ratio of breast milk OCP residues and their associations to women with average menstrual period days of >5 days as determined by logistic regression models
Table S18
Odds ratio of breast milk OCP residues and their associations to women with the shortest menstrual period days of ≤3 days as determined by logistic regression models
Table S19
Odds ratio of breast milk OCP residues and their associations to women who have taken contraceptives as determined by logistic regression models
Table S20
Odds ratio of breast milk OCP residues and their associations to women who have or have not taken hormonal drugs as determined by logistic regression models
Table S21
Odds ratios of OCP residues in breast milk from mothers who received infertility medical treatment in comparison to normal mothers as determined by logistic regression models
Table S22
Odds ratios of breast milk OCP residues in the participants having undergone gynecological surgery compared with those in normal women as determined by logistic regression models
Table S23
Sociodemographic characteristics, dietary habits and menstruation characteristics of mothers in association with OCP residues in breast milk as determined using the logistic regression model
Text: Limitation of small sample size

Table S1. Breast milk OCP levels (ng/g lipid) of Taiwanese women who were born before, in, and after 1975

	Before and in 1975 (n = 27)	After 1975 year (n = 41)	*p*-vale
Aldrin	0.234±0.594	0.134±0.302	0.099 [#]
ΣHCH[a]	0.619±0.599	0.491±0.524	0.182
α- HCH	0.147±0.296	0.123±0.286	0.401
β- HCH	0.146±0.209	0.104±0.237	0.131
γ- HCH	0.112±0.133	0.0798±0.0757	0.140
δ- HCH	0.0956±0.135	0.0938±0.101	0.940
ΣCHL[a]	0.193±0.258	0.141±0.291	0.119
cis-Chlordane (cis-CHL)	0.103±0.167	0.0594±0.0591	0.026*
trans-Chlordane (trans-CHL)	0.0570±0.151	0.0560±0.294	0.952
ΣDDT[a]	9.79±5.31	9.81±8.70	0.817
4,4- DDD	0.197±1.315	0.140±1.83	0.451
4,4- DDE	8.04±4.87	8.09±7.46	0.684
4,4- DDT	0.388±0.609	0.341±0.898	0.915
Dieldrin	0.181±0.497	0.162±0.503	0.721
ΣEndosulfan[a]	0.335±0.575	0.263±0.881	0.290
Endosulfan I	0.0837±0.263	0.0927±0.431	0.843
Endosulfan II	0.0554±0.351	0.0462±0.393	0.769
Endosulfan sulfate	0.0939±0.192	0.0595±0.148	0.149
ΣEndrin[a]	0.418±0.543	0.358±0.788	0.357
Endrin	0.176±0.307	0.176±0.503	0.535
Endrin aldehyde	0.0675±0.240	0.0583±0.272	0.615
Endrin ketone	0.0632±0.178	0.0570±0.172	0.764
ΣHeptachlor[a]	0.695±0.945	0.613±1.036	0.730
Heptachlor	0.423±0.656	0.348±0.679	0.511
Heptachlor epoxide (isomer B)	0.217±0.329	0.216±0.403	0.925
Methoxychlor	0.0500±0.150	0.0329±0.132	0.265

[a] ΣHCH is the sum of α, β, γ, and δ- HCH; ΣCHL is the sum of cis- and trans-CHL; ΣDDT is the sum of 4,4'-DDD, 4,4'-DDE, and 4,4'-DDT; ΣEndosulfan is the sum of endosulfanI, endosulfan II, and endosulfan sulfate; ΣEndrin is the sum of endrin, endrinaldehyde, and endrinketone; ΣHeptachlor is the sum of heptachlor and heptachlor epoxide.
* $p < 0.05$, [#] $p < 0.1$.

Table S2. Odds ratios of OCP residues in breast milk and their correlation to native-born Aborigines (n = 12) in comparison to native-born and nonnative-born Taiwanese (n = 56) as determined by the logistic regression model

OCPs	Odds Ratio[a]	95% confidence intervals	*p*-value
Log Aldrin	5.63	0.852-37.2	0.073[#]
Log α- HCH	0.348	0.036-3.34	0.360
Log β- HCH	2.17	0.288-16.4	0.452
Log γ- HCH	0.659	0.061-7.17	0.732
Log δ- HCH	0.331	0.030-3.66	0.368
Log ΣHCH	0.944	0.040-22.5	0.972
Log cis-CHL	2.31	0.233-22.8	0.476
Log trans-CHL	6.48	0.890-47.1	0.065[#]
Log ΣCHL	13.2	0.706-246	0.084[#]
Log 4,4′-DDD	0.718	0.302-1.71	0.453
Log 4,4′-DDE	0.666	0.036-12.4	0.785
Log 4,4′-DDT	2.09	0.439-9.94	0.354
Log ΣDDT	0.442	0.015-12.8	0.634
Log Dieldrin	0.772	0.141-4.23	0.765
Log Endosulfan I	0.741	0.133-4.14	0.732
Log Endosulfan II	0.904	0.186-4.40	0.900
Log Endosulfan sulfate	10.8	1.03-113	0.047*
Log ΣEndosulfan	1.08	0.131-8.96	0.942
Log Endrin	0.531	0.057-4.91	0.577
Log Endrin aldehyde	0.435	0.078-2.43	0.343
Log Endrin ketone	0.784	0.140-4.38	0.782
Log ΣEndrin	0.434	0.0385-5.01	0.504
Log Heptachlor	1.36	0.224-8.24	0.740
Log Heptachlor epoxide (isomer B)	2.21	0.367-13.3	0.387
Log ΣHeptachlor	2.12	0.286-15.7	0.462
Log Methoxychlor	0.907	0.215-3.82	0.894

[#] $p < 0.1$, * $p < 0.05$.

[a]Adjusted by pre-pregnant BMI, age, annual income, birth year and parity.

Table S3. Odds ratios of OCP residues in breast milk and their correlation to women who were born before and during 1975 (n = 27) in comparison to women born after 1975 (n = 41) as determined by the logistic regression model

OCPs	Odds Ratio[a]	95% confidence intervals	*p*-value
Log Aldrin	1.79	0.192-16.7	0.610
Log α- HCH	2.65	0.134-52.1	0.522
Log β- HCH	7.28	0.408-130	0.177
Log γ- HCH	2.54	0.100-64.5	0.573
Log δ- HCH	334	0.111-1004566	0.155
Log ΣHCH	36.7	0.338-3987	0.132
Log cis-CHL	6.45	0.205-203	0.289
Log trans-CHL	0.025	0.0001-3.93	0.153
Log ΣCHL	0.388	0.007-20.5	0.640
Log 4,4′-DDD	0.322	0.050-2.09	0.235
Log 4,4′-DDE	3.88	0.061-247	0.522
Log 4,4′-DDT	2.61	0.189-36.1	0.474
Log ΣDDT	2.38	0.042-134	0.674
Log Dieldrin	0.701	0.081-6.06	0.747
Log Endosulfan I	0.152	0.007-3.50	0.239
Log Endosulfan II	0.973	0.145-6.53	0.978
Log Endosulfan sulfate	2.40	0.261-22.1	0.439
Log ΣEndosulfan	0.532	0.037-7.55	0.641
Log Endrin	0.071	0.001-4.15	0.202
Log Endrin aldehyde	0.186	0.011-3.07	0.240
Log Endrin ketone	0.500	0.052-4.80	0.548
Log ΣEndrin	0.067	0.001-3.26	0.173
Log Heptachlor	0.095	0.002-4.49	0.232
Log Heptachlor epoxide (isomer B)	0.0615	0.065-5.78	0.671
Log ΣHeptachlor	0.061	0.001-3.377	0.172
Log Methoxychlor	1.14	0.145-8.91	0.904

[a]Adjusted by pre-pregnant BMI, age, population, annual income, and parity.

Table S4. Odds ratios of OCP residues in breast milk and their correlation to older mothers (>31 years old) in comparison to younger mothers (≤31 years old) as determined by the logistic regression model

OCPs	Odds Ratio[a]	95% confidence intervals	*p*-value
Log Aldrin	4.29	0.117-157	0.428
Log α- HCH	0.230	0.003-17.4	0.506
Log β- HCH	0.023	0.0001-9.45	0.219
Log γ- HCH	-	-	-
Log δ- HCH	0.265	0.006-11.0	0.485
Log ΣHCH	-	-	-
Log cis-CHL	0.017	0.0001-30.4	0.287
Log trans-CHL	194	0.015-2564221	0.276
Log ΣCHL	18.3	0.126-2667	0.252
Log 4,4′-DDD	0.952	0.212-4.28	0.949
Log 4,4′-DDE	7.60	0.003-19771	0.613
Log 4,4′-DDT	0.925	0.072-12.0	0.953
Log ΣDDT	8.54	0.003-21179	0.591
Log Dieldrin	2.48	0.098-63.0	0.582
Log Endosulfan I	1.16	0.078-17.1	0.916
Log Endosulfan II	-	-	-
Log Endosulfan sulfate	0.356	0.014-9.00	0.531
Log ΣEndosulfan	0.147	0.001-20.6	0.447
Log Endrin	3.55	0.073-172	0.522
Log Endrin aldehyde	1.56	0.09*2-26.2	0.759
Log Endrin ketone	0.319	0.006-17.8	0.577
Log ΣEndrin	1.67	0.024-114	0.812
Log Heptachlor	9.77	0.134-713	0.296
Log Heptachlor epoxide (isomer B)	0.257	0.008-8.56	0.447
Log ΣHeptachlor	6.43	0.106-390	0.374
Log Methoxychlor	-	-	-

[a] Adjusted by pre-pregnant BMI, population, annual income, birth year and parity.

Table S5. Odds ratios of OCP residues in breast milk and their correlation to mothers who have higher pre-pregnant BMI values (>21.7 kg m^{-2}) in comparison to those having lower pre-pregnant BMI values (≤21.7 kg m^{-2}) as determined by the logistic regression model

OCPs	Odds Ratio[a]	95% confidence intervals	*p*-value
Log Aldrin	1.49	0.492-4.50	0.480
Log α- HCH	1.10	0.351-3.43	0.872
Log β- HCH	1.08	0.302-3.85	0.908
Log γ- HCH	1.35	0.335-5.41	0.676
Log δ- HCH	0.457	0.124-1.69	0.239
Log ΣHCH	1.04	0.160-6.75	0.967
Log cis-CHL	1.33	0.351-5.06	0.674
Log trans-CHL	1.19	0.425-3.33	0.740
Log ΣCHL	1.26	0.295-5.38	0.756
Log 4,4′-DDD	1.24	0.766-1.99	0.385
Log 4,4′-DDE	0.540	0.120-2.42	0.420
Log 4,4′-DDT	0.478	0.201-1.14	0.095[#]
Log ΣDDT	0.482	0.090-2.57	0.393
Log Dieldrin	1.60	0.632-4.04	0.322
Log Endosulfan I	1.28	0.521-3.15	0.589
Log Endosulfan II	1.32	0.582-2.98	0.510
Log Endosulfan sulfate	1.56	0.599-4.05	0.363
Log ΣEndosulfan	2.11	0.685-6.55	0.193
Log Endrin	1.18	0.388-3.56	0.775
Log Endrin aldehyde	1.52	0.647-3.59	0.336
Log Endrin ketone	1.21	0.463-3.17	0.696
Log ΣEndrin	1.85	0.513-6.64	0.349
Log Heptachlor	2.81	0.924-8.56	0.069[#]
Log Heptachlor epoxide (isomer B)	1.49	0.544-4.10	0.436
Log ΣHeptachlor	2.52	0.746-8.52	0.136
Log Methoxychlor	1.75	0.731-4.20	0.208

[#] $p < 0.1$.

[a] Adjusted by age, population, annual income, birth year and parity.

Table S6. Odds ratio of OCP residues in breast milk and their correlation to mothers having low annual family income (≤$20,000 US dollar) in comparison to mothers having high annual family income (>$20,000 US dollar) as determined by the logistic regression model

OCPs	Odds Ratio[a]	95% confidence intervals	*p*-value
Log Aldrin	2.70	0.958-7.61	0.060#
Log α- HCH	4.20	1.08-16.2	0.037*
Log β- HCH	3.46	0.907-13.2	0.069
Log γ- HCH	1.10	0.258-4.65	0.903
Log δ- HCH	1.92	0.504-7.32	0.339
Log ΣHCH	10.7	1.27-90.1	0.029*
Log cis-CHL	0.918	0.233-3.61	0.902
Log trans-CHL	0.866	0.294-2.55	0.793
Log ΣCHL	1.02	0.224-4.60	0.984
Log 4,4′-DDD	1.19	0.718-1.98	0.495
Log 4,4′-DDE	4.06	0.706-23.3	0.116
Log 4,4′-DDT	0.824	0.345-1.97	0.663
Log ΣDDT	4.15	0.628-27.4	0.140
Log Dieldrin	1.78	0.662-4.80	0.253
Log Endosulfan I	0.898	0.354-2.27	0.820
Log Endosulfan II	1.07	0.453-2.52	0.880
Log Endosulfan sulfate	1.43	0.538-3.78	0.475
Log ΣEndosulfan	1.38	0.431-4.42	0.587
Log Endrin	1.418	0.446-4.51	0.554
Log Endrin aldehyde	1.61	0.659-3.92	0.298
Log Endrin ketone	0.700	0.253-1.94	0.493
Log ΣEndrin	1.67	0.437-6.36	0.454
Log Heptachlor	1.71	0.532-5.49	0.368
Log Heptachlor epoxide (isomer B)	1.12	0.398-3.15	0.830
Log ΣHeptachlor	1.66	0.470-5.84	0.432
Log Methoxychlor	1.73	0.689-4.35	0.243

[#] $p < 0.1$, * $p < 0.05$.

[a] Adjusted by pre-pregnant BMI, age, population, birth year and parity.

Table S7. Odds ratio of OCP residues in breast milk and their correlation to primiparous mothers in comparison to mothers who are multiparous as determined by the logistic regression model

OCPs	Odds Ratio[a]	95% confidence intervals	*p*-value
Log Aldrin	1.51	0.427-5.33	0.523
Log α- HCH	3.85	0.714-20.8	0.117
Log β- HCH	5.14	0.884-29.9	0.068[#]
Log γ- HCH	0.820	0.121-5.55	0.839
Log δ- HCH	3.69	0.608-22.4	0.156
Log ΣHCH	27.3	1.63-457	0.021*
Log cis-CHL	0.768	0.105-5.63	0.795
Log trans-CHL	1.98	0.468-8.42	0.353
Log ΣCHL	3.71	0.485-28.4	0.207
Log 4,4′-DDD	0.895	0.465-1.72	0.740
Log 4,4′-DDE	0.813	0.137-4.83	0.820
Log 4,4′-DDT	0.992	0.350-2.81	0.987
Log ΣDDT	0.779	0.107-5.68	0.805
Log Dieldrin	0.199	0.038-1.04	0.056[#]
Log Endosulfan I	1.14	0.334-3.89	0.834
Log Endosulfan II	0.813	0.242-2.73	0.737
Log Endosulfan sulfate	0.855	0.221-3.32	0.821
Log ΣEndosulfan	0.948	0.207-4.34	0.945
Log Endrin	1.64	0.362-7.38	0.523
Log Endrin aldehyde	1.93	0.573-6.51	0.288
Log Endrin ketone	0.325	0.070-1.51	0.152
Log ΣEndrin	1.42	0.271-7.47	0.676
Log Heptachlor	1.87	0.436-8.00	0.400
Log Heptachlor epoxide (isomer B)	1.87	0.432-8.06	0.403
Log ΣHeptachlor	1.81	0.368-8.89	0.465
Log Methoxychlor	1.19	0.347-4.09	0.782

[#] $p < 0.1$, * $p < 0.05$.

[a] Adjusted by pre-pregnant BMI, age, population, annual income, and birth year.

Table S8. Odds ratio of OCP residues in breast milk and their correlation to mothers who have attained low education levels (pre-senior and senior high school) in comparison to those who with high education background (tertiary education and graduate education) as determined by the logistic regression model

OCPs	Odds Ratio[a]	95% confidence intervals	*p*-value
Log Aldrin	0.522	0.172-1.58	0.251
Log α- HCH	1.96	0.530-7.27	0.313
Log β- HCH	2.67	0.554-12.8	0.221
Log γ- HCH	0.251	0.043-1.48	0.127
Log δ- HCH	2.20	0.545-8.92	0.268
Log ΣHCH	2.54	0.281-23.0	0.407
Log cis-CHL	1.08	0.236-4.94	0.920
Log trans-CHL	0.703	0.227-2.18	0.541
Log ΣCHL	1.23	0.246-6.17	0.800
Log 4,4′-DDD	1.02	0.583-1.80	0.937
Log 4,4′-DDE	0.988	0.192-5.09	0.989
Log 4,4′-DDT	1.68	0.628-4.50	0.301
Log ΣDDT	1.16	0.187-7.17	0.875
Log Dieldrin	0.998	0.344-2.89	0.997
Log Endosulfan I	1.01	0.353-2.87	0.992
Log Endosulfan II	0.814	0.326-2.04	0.659
Log Endosulfan sulfate	0.622	0.193-2.00	0.426
Log ΣEndosulfan	0.912	0.256-3.24	0.887
Log Endrin	1.01	0.286-3.55	0.991
Log Endrin aldehyde	1.59	0.591-4.26	0.359
Log Endrin ketone	1.826	0.560-5.96	0.318
Log ΣEndrin	1.59	0.341-7.40	0.556
Log Heptachlor	0.718	0.186-2.77	0.630
Log Heptachlor epoxide (isomer B)	0.771	0.229-2.59	0.674
Log ΣHeptachlor	0.743	0.169-3.27	0.695
Log Methoxychlor	1.02	0.378-2.76	0.967

[a] Adjusted by pre-pregnant BMI, age, population, annual income, birth year and parity.

Table S9. Associations of poultry (egg) and dairy products (milk and cheese) consumption to breastmilk OCPs as determined by Mann-Whitney *U* Test

	Milk consumption (mL/ week)			Cheese consumption (piece/ week)			Egg consumption (piece/ week)		
	≤ 625 (n=51)	> 625 (n=17)	*p*-value	≤ 1 (n=60)	> 1(n=8)	*p*-value	≤ 5 (n=38)	> 5 (n=30)	*p*-value
	GM±GSD [a]	GM±GSD		GM±GSD	GM±GSD		GM±GSD	GM±GSD	
Aldrin	0.147±3.89	0.247±3.85	0.114	0.171±4.14	0.145±2.47	0.634	0.182±3.76	0.151±4.17	0.657
ΣHCH[a]	0.503±1.95	0.661±1.92	0.075	0.534±1.96	0.577±1.97	0.732	0.541±1.81	0.536±2.14	0.882
α- HCH	0.124±2.69	0.164±3.02	0.231	0.135±2.64	0.118±4.08	0.746	0.148±2.46	0.115±3.16	0.212
β- HCH	0.104±2.63	0.185±2.57	0.015*	0.117±2.75	0.142±2.24	0.634	0.113±2.40	0.129±3.07	0.753
γ- HCH	0.0879±2.21	0.103±2.82	0.192	0.0899±2.35	0.104±2.46	0.499	0.0998±2.29	0.0818±2.43	0.643
δ- HCH	0.0980±2.61	0.0847±2.80	0.707	0.0963±2.61	0.0823±3.01	0.970	0.0873±2.71	0.105±2.57	0.488
ΣCHL[a]	0.140±2.22	0.243±2.24	0.010*	0.153±2.26	0.229±2.50	0.278	0.150±2.23	0.174±2.39	0.545
cis-CHL	0.0703±2.32	0.0860±3.46	0.421	0.0698±2.56	0.114±2.62	0.566	0.0728±2.65	0.0754±2.55	0.813
trans-CHL	0.0456±3.08	0.106±2.67	0.004**	0.0525±2.95	0.0966±2.92	0.075	0.0532±2.94	0.0607±3.44	0.694
ΣDDT[a]	9.47±2.02	10.9±1.88	0.221	10.2±1.84	7.04±3.02	0.621	9.53±1.75	10.2±2.28	0.401
4,4- DDD	0.121±10.8	0.382±9.98	0.127	0.161±10.7	0.161±14.8	0.984	0.154±11.2	0.170±11.0	0.974
4,4- DDE	7.82±2.18	8.87±2.10	0.318	8.57±1.95	5.12±3.49	0.332	7.80±1.87	8.42±2.51	0.261
4,4- DDT	0.413±4.21	0.237±3.61	0.065	0.354±3.89	0.401±6.49	0.661	0.327±3.98	0.406±4.32	0.485
Dieldrin	0.161±3.63	0.200±3.76	0.254	0.183±3.60	0.0959±3.72	0.253	0.173±3.63	0.166±3.72	0.990
ΣEndosulfan[a]	0.240±2.82	0.511±2.43	0.006*	0.296±2.84	0.247±3.17	0.481	0.273±2.64	0.312±3.18	0.824
Endosulfan I	0.0714±3.60	0.172±3.13	0.014*	0.0892±3.67	0.0875±3.89	0.954	0.0798±3.27	0.102±4.20	0.650
Endosulfan II	0.0382±3.79	0.109±4.05	0.005**	0.0492±3.96	0.0536±5.93	0.934	0.0383±3.69	0.0691±4.48	0.065
Endosulfan sulfate	0.0774±2.96	0.0556±5.29	0.605	0.0800±3.39	0.0300±3.27	0.039*	0.0893±3.16	0.0536±3.78	0.130
ΣEndrin[a]	0.318±2.44	0.655±2.22	0.002*	0.373±2.52	0.444±2.57	0.864	0.365±2.38	0.403±2.71	0.990
Endrin	0.149±2.89	0.294±2.44	0.011*	0.174±3.00	0.194±2.04	0.819	0.171±2.83	0.183±2.99	0.902
Endrin aldehyde	0.0530±3.56	0.0979±4.99	0.123	0.0565±3.84	0.122±4.52	0.216	0.0528±3.88	0.0755±4.05	0.292
Endrin ketone	0.0482±3.31	0.111±3.22	0.012*	0.0590±3.44	0.0625±3.77	0.856	0.0569±3.54	0.0626±3.38	0.675
ΣHeptachlor[a]	0.537±2.69	1.11±2.22	0.007**	0.660±2.75	0.543±2.33	0.581	0.713±2.57	0.567±2.86	0.348
Heptachlor	0.305±3.03	0.705±2.34	0.007**	0.387±3.04	0.309±3.03	0.594	0.416±2.81	0.331±3.30	0.408
Heptachlor epoxide (isomer B)	0.203±2.66	0.265±4.58	0.133	0.234±2.93	0.123±4.26	0.253	0.234±3.30	0.197±2.90	0.320
Methoxychlor	0.0353±3.73	0.0516±4.32	0.293	0.0396±3.88	0.0336±4.09	0.797	0.0339±4.16	0.0461±3.58	0.213

[a] Geometric mean ± geometric standard deviation.

Table S10. Meat (beef, pork, and chicken) consumption in correlation to breast milk OCPs as determined by Mann-Whitney *U* Test

	Beef consumption (gram/week)			Pork consumption (gram/week)			Chicken consumption (gram/week)		
	≤ 50 (n=55)	> 50 (n=13)	*p*-value	≤ 250 (n=44)	> 250(n=24)	*p*-value	≤ 125 (n=36)	> 125 (n=32)	*p*-value
	GM±GSD	GM±GSD		GM±GSD	GM±GSD		GM±GSD	GM±GSD	
Aldrin	0.150±3.81	0.267±4.20	0.124	0.171±4.18	0.162±3.55	0.768	0.174±4.75	0.161±3.10	0.796
ΣHCH[a]	0.541±1.99	0.531±1.82	0.932	0.557±1.92	0.507±2.04	0.419	0.544±2.19	0.532±1.69	0.892
α- HCH	0.138±2.84	0.113±2.57	0.797	0.142±2.76	0.118±2.83	0.287	0.125±3.19	0.141±2.34	0.403
β- HCH	0.115±2.77	0.145±2.34	0.449	0.123±2.61	0.114±2.87	0.488	0.119±3.12	0.120±2.23	0.995
γ- HCH	0.0986±2.19	0.0665±2.96	0.378	0.104±2.25	0.0718±2.46	0.138	0.0888±2.59	0.0945±2.11	0.782
δ- HCH	0.0969±2.48	0.0853±3.47	0.845	0.0946±2.55	0.0944±2.88	0.995	0.102±2.62	0.0865±2.69	0.486
ΣCHL[a]	0.141±2.22	0.275±2.19	0.010*	0.159±2.22	0.163±2.47	0.748	0.166±2.33	0.154±2.28	0.787
cis-CHL	0.0646±2.54	0.131±2.31	0.008*	0.0788±2.56	0.0659±2.66	0.230	0.0755±2.67	0.0723±2.53	0.834
trans-CHL	0.0488±2.93	0.104±3.58	0.028*	0.0533±3.03	0.0625±3.40	0.703	0.0529±3.27	0.0606±3.04	0.433
ΣDDT[a]	9.69±2.10	10.3±1.43	0.845	9.81±1.87	9.80±2.21	0.888	9.98±2.03	9.62±1.95	0.941
4,4- DDD	0.131±11.3	0.382±8.85	0.138	0.197±11.7	0.111±9.91	0.301	0.155±12.6	0.167±9.76	0.903
4,4- DDE	8.01 ±2.30	8.29±1.52	0.612	8.00±2.01	8.20±2.44	0.739	8.07±2.18	8.06±2.15	0.825
4,4- DDT	0.367±4.07	0.328±4.50	0.773	0.294±4.10	0.519±3.92	0.117	0.479±3.43	0.260±4.69	0.095
Dieldrin	0.165±3.69	0.191±3.61	0.612	0.196±3.74	0.131±3.41	0.253	0.174±4.48	0.165±2.81	0.912
ΣEndosulfan[a]	0.256±2.84	0.491±2.55	0.060	0.291±2.74	0.288±3.14	0.739	0.277±3.23	0.305±2.48	0.658
Endosulfan I	0.0872±3.76	0.0972±3.42	0.732	0.0919±3.48	0.0841±4.12	0.565	0.0960±4.36	0.0818±2.97	0.783
Endosulfan II	0.0424±3.80	0.0973±4.95	0.078	0.0422±3.74	0.0671±4.75	0.183	0.0416±4.31	0.0607±3.89	0.207
Endosulfan sulfate	0.0609±3.38	0.138±3.38	0.024*	0.0783±3.67	0.0600±3.21	0.361	0.0598±3.62	0.0869±3.32	0.150
ΣEndrin[a]	0.365±2.58	0.459±2.24	0.314	0.382±2.39	0.380±2.79	0.457	0.393±2.55	0.368±2.50	0.854
Endrin	0.181±3.00	0.160±2.45	0.981	0.175±2.74	0.179±3.21	0.599	0.201±2.81	0.152±2.96	0.491
Endrin aldehyde	0.0637±3.79	0.0544±4.96	0.746	0.0613±3.71	0.0628±4.57	0.835	0.0675±3.74	0.0560±4.28	0.600
Endrin ketone	0.0503±3.21	0.120±3.75	0.026*	0.0583±3.76	0.0615±2.95	0.682	0.0477±3.45	0.0761±3.34	0.087
ΣHeptachlor[a]	0.595±2.69	0.902±2.63	0.145	0.694±2.77	0.564±2.58	0.355	0.604±2.98	0.694±2.41	0.531
Heptachlor	0.347±3.00	0.528±3.06	0.221	0.412±3.08	0.319±2.93	0.287	0.357±3.31	0.399±2.75	0.731
Heptachlor epoxide (isomer B)	0.197±3.19	0.326±2.57	0.128	0.214±3.54	0.221±2.38	0.748	0.194±3.51	0.245±2.67	0.465
Methoxychlor	0.0334±3.83	0.0732±3.55	0.063	0.0377±4.13	0.0410±3.52	0.602	0.0396±4.34	0.0380±3.45	0.936

[a] Geometric mean ± geometric standard deviation.

Table S11. Odds ratios of OCP residues in breast milk and their correlation to cow milk consumption as determined by logistic regression models

OCPs	Odds Ratio[a]	95% confidence intervals	*p*-value
Log Aldrin	2.73	0.843-8.83	0.094
Log β- HCH	7.35	1.45-37.3	0.016*
Log ΣHCH	7.35	0.894-60.3	0.064
Log ΣCHL	6.65	1.10-40.1	0.039*
Log 4,4′-DDE	1.88	0.302-11.7	0.499
Log 4,4′-DDT	0.526	0.198-1.40	0.197
Log Dieldrin	1.56	0.528-4.629	0.420
Log Endosulfan I	3.03	1.04-8.83	0.043*
Log Endosulfan II	4.53	1.56-13.1	0.005**
Log ΣEndosulfan	6.67	1.53-28.9	0.011*
Log Endrin	6.73	1.32-34.4	0.022*
Log Endrin ketone	3.72	1.18-11.7	0.025*
Log ΣEndrin	13.3	2.05-86.1	0.007**
Log ΣHeptachlor	9.11	1.72-48.4	0.010*
Log Methoxychlor	2.02	0.722-5.64	0.181

[a]Adjusted by pre-pregnant BMI, age, population, annual income, birth year and parity.

* $p < 0.05$.

Table S12. Odds ratios of OCP residues in breast milk and their correlation to beef consumption as determined by logistic regression models

OCPs	Odds Ratio[a]	95% confidence intervals	*p*-value
Log Aldrin	2.43	0.612-9.62	0.207
Log α- HCH	0.765	0.150-3.90	0.747
Log β- HCH	2.37	0.459-12.3	0.303
Log γ- HCH	0.128	0.017-0.946	0.044*
Log δ- HCH	0.566	0.108-2.96	0.500
Log ΣHCH	1.15	0.100-13.2	0.911
Log cis-CHL	5.64	0.741-42.8	0.095#
Log trans-CHL	5.05	1.25-20.4	0.023*
Log ΣCHL	10.5	1.36-81.2	0.024*
Log 4,4′-DDD	1.71	0.848-3.46	0.134
Log 4,4′-DDE	1.10	0.133-9.10	0.928
Log 4,4′-DDT	0.870	0.268-2.83	0.818
Log ΣDDT	1.29	0.108-15.2	0.843
Log Dieldrin	1.37	0.384-4.89	0.627
Log Endosulfan I	0.965	0.259-3.60	0.957
Log Endosulfan II	4.18	1.27-13.8	0.019*
Log Endosulfan sulfate	7.16	1.36-37.7	0.020*
Log ΣEndosulfan	6.88	1.24-38.0	0.027*
Log Endrin	0.692	0.138-3.46	0.654
Log Endrin aldehyde	0.865	0.268-2.79	0.808
Log Endrin ketone	3.68	0.957-14.2	0.058#
Log ΣEndrin	2.35	0.384-14.4	0.356
Log Heptachlor	3.67	0.721-18.6	0.117
Log Heptachlor epoxide (isomer B)	4.11	0.721-23.4	0.111
Log ΣHeptachlor	4.70	0.769-28.8	0.094#
Log Methoxychlor	4.01	1.13-14.2	0.032*

[a] Adjusted by pre-pregnant BMI, age, population, annual income, birth year and parity.

* $p < 0.05$, # $p < 0.1$.

Table S13. Odds ratios of OCP residues in breast milk and their correlation to pork consumption as determined by logistic regression models

OCPs	Odds Ratio[a]	95% confidence intervals	*p*-value
Log Aldrin	1.24	0.455-3.40	0.671
Log α- HCH	0.881	0.243-3.19	0.847
Log β- HCH	1.52	0.405-5.67	0.537
Log γ- HCH	0.300	0.060-1.50	0.142
Log δ- HCH	1.09	0.270-4.40	0.903
Log ΣHCH	1.28	0.184-8.82	0.806
Log cis-CHL	0.426	0.090-2.02	0.283
Log trans-CHL	1.35	0.430-4.20	0.610
Log ΣCHL	1.11	0.214-5.73	0.904
Log 4,4′-DDD	0.782	0.453-1.35	0.378
Log 4,4′-DDE	1.32	0.269-6.51	0.731
Log 4,4′-DDT	2.58	0.905-7.33	0.076[#]
Log ΣDDT	1.15	0.193-6.86	0.878
Log Dieldrin	0.575	0.197-1.68	0.312
Log Endosulfan I	0.744	0.259-2.14	0.584
Log Endosulfan II	2.19	0.869-5.53	0.096[#]
Log Endosulfan sulfate	0.780	0.269-2.26	0.646
Log ΣEndosulfan	1.17	0.333-4.10	0.807
Log Endrin	1.24	0.348-4.44	0.737
Log Endrin aldehyde	1.23	0.473-3.19	0.673
Log Endrin ketone	0.874	0.298-2.56	0.807
Log ΣEndrin	1.18	0.288-4.81	0.820
Log Heptachlor	0.681	0.201-2.31	0.537
Log Heptachlor epoxide (isomer B)	1.10	0.342-3.56	0.869
Log ΣHeptachlor	0.673	0.176-2.58	0.564
Log Methoxychlor	1.58	0.576-4.32	0.375

[a] Adjusted by pre-pregnant BMI, age, population, annual income, birth year and parity.

[#] $p < 0.1$.

Table S14. Odds ratios of OCP residues in breast milk and their correlation to chicken consumption as determined by logistic regression models

OCPs	Odds Ratio[a]	95% confidence intervals	*p*-value
Log Aldrin	0.854	0.344-2.12	0.734
Log α- HCH	1.51	0.470-4.87	0.487
Log β- HCH	1.24	0.366-4.23	0.727
Log γ- HCH	1.33	0.325-5.41	0.693
Log δ- HCH	0.637	0.191-2.13	0.464
Log ΣHCH	1.10	0.186-6.44	0.920
Log cis-CHL	0.676	0.172-2.66	0.575
Log trans-CHL	1.29	0.458-3.62	0.631
Log ΣCHL	0.640	0.143-2.87	0.560
Log 4,4′-DDD	1.01	0.623-1.65	0.956
Log 4,4′-DDE	1.10	0.247-4.87	0.903
Log 4,4′-DDT	0.475	0.196-1.15	0.098[#]
Log ΣDDT	0.905	0.170-4.82	0.907
Log Dieldrin	0.913	0.359-2.32	0.848
Log Endosulfan I	0.695	0.270-1.79	0.450
Log Endosulfan II	1.63	0.710-3.73	0.250
Log Endosulfan sulfate	2.00	0.749-5.34	0.167
Log ΣEndosulfan	1.19	0.385-3.69	0.760
Log Endrin	0.496	0.155-1.59	0.237
Log Endrin aldehyde	0.822	0.347-1.94	0.822
Log Endrin ketone	1.88	0.723-4.87	0.196
Log ΣEndrin	0.797	0.219-2.91	0.731
Log Heptachlor	1.26	0.416-3.79	0.687
Log Heptachlor epoxide (isomer B)	1.54	0.540-4.39	0.419
Log ΣHeptachlor	1.46	0.433-4.93	0.541
Log Methoxychlor	0.952	0.393-2.31	0.914

[a] Adjusted by pre-pregnant BMI, age, population, annual income, birth year and parity.

[#] $p < 0.1$.

Table S15. Odds ratios of OCP residues in breast milk and their associations to mothers who menarche before 13 years old in comparison to mothers who menarche after 13 years old as determined by logistic regression models

OCPs	Odds Ratio[a]	95% confidence intervals	*p*-value
Log Aldrin	0.679	0.197-2.34	0.539
Log α- HCH	0.937	0.246-3.57	0.924
Log β- HCH	0.324	0.059-1.80	0.324
Log γ- HCH	0.186	0.028-1.25	0.083
Log δ- HCH	1.58	0.331-7.56	0.565
Log ΣHCH	0.297	0.026-3.34	0.326
Log cis-CHL	0.164	0.023-1.18	0.073
Log trans-CHL	0.836	0.250-2.79	0.770
Log ΣCHL	0.530	0.095-2.96	0.470
Log 4,4′-DDD	0.770	0.388-1.53	0.456
Log 4,4′-DDE	1.77	0.301-10.4	0.527
Log 4,4′-DDT	2.05	0.647-6.52	0.222
Log ΣDDT	1.72	0.234-12.7	0.593
Log Dieldrin	0.325	0.088-1.20	0.093
Log Endosulfan I	0.593	0.186-1.89	0.377
Log Endosulfan II	2.52	0.833-7.64	0.101
Log Endosulfan sulfate	1.80	0.601-5.37	0.294
Log ΣEndosulfan	1.28	0.285-5.76	0.747
Log Endrin	0.285	0.064-1.26	0.098
Log Endrin aldehyde	0.466	0.163-1.33	0.153
Log Endrin ketone	0.829	0.248-2.77	0.760
Log ΣEndrin	0.385	0.074-2.02	0.259
Log Heptachlor	0.537	0.110-2.61	0.440
Log Heptachlor epoxide (isomer B)	1.26	0.340-4.65	0.731
Log ΣHeptachlor	0.572	0.106-3.09	0.516
Log Methoxychlor	0.654	0.208-2.06.	0.654

[a] Adjusted by pre-pregnant BMI, age, population, annual income, birth year and parity.

Table S16. Odds ratio of average periods of menstrual cycles of 27 to 29 days as compared to menstrual cycles of ≤ 26 and ≥ 30 days as determined by logistic regression models

OCPs	Odds Ratio[a]	95% confidence intervals	*p*-value
Log Aldrin	0.926	0.131-2.74	0.890
Log α- HCH	0.392	0.102-1.51	0.173
Log β- HCH	1.00	0.220-4.54	0.999
Log γ- HCH	2.85	0.565-14.3	0.205
Log δ- HCH	1.82	0.436-7.56	0.413
Log ΣHCH	0.625	0.070-5.58	0.674
Log cis-CHL	1.16	0.240-5.56	0.857
Log trans-CHL	0.985	0.306-3.17	0.980
Log ΣCHL	1.00	0.189-5.28	1.00
Log 4,4′-DDD	0.826	0.454-1.50	0.532
Log 4,4′-DDE	2.94	0.469-18.4	0.250
Log 4,4′-DDT	2.14	0.761-6.02	0.149
Log ΣDDT	3.31	0.438-25.0	0.246
Log Dieldrin	0.873	0.303-2.52	0.802
Log Endosulfan I	0.668	0.237-1.88	0.444
Log Endosulfan II	0.409	0.157-1.07	0.067
Log Endosulfan sulfate	0.810	0.290-2.27	0.688
Log ΣEndosulfan	0.340	0.083-1.39	0.134
Log Endrin	2.04	0.534-7.75	0.298
Log Endrin aldehyde	1.17	0.445-3.08	0.750
Log Endrin ketone	0.397	0.131-1.20	0.102
Log ΣEndrin	0.710	0.161-3.14	0.651
Log Heptachlor	0.843	0.192-3.69	0.820
Log Heptachlor epoxide (isomer B)	0.698	0.220-2.22	0.542
Log ΣHeptachlor	0.761	0.158-3.66	0.734
Log Methoxychlor	0.515	0.178-1.49	0.220

[a] Adjusted by pre-pregnant BMI, age, population, annual income, birth year and parity.

Table S17. Odds ratio of breast milk OCP residues and their associations to women with average menstrual period days of >5 days as determined by logistic regression models

OCPs	Odds Ratio[a]	95% confidence intervals	*p*-value
Log Aldrin	1.55	0.473-5.11	0.468
Log α- HCH	1.26	0.333-4.80	0.731
Log β- HCH	0.609	0.1174-3.16	0.555
Log γ- HCH	0.269	0.047-1.54	0.140
Log δ- HCH	1.26	0.285-5.56	0.761
Log ΣHCH	0.428	0.042-4.40	0.476
Log cis-CHL	1.02	0.195-5.27	0.986
Log trans-CHL	4.73	1.12-20.1	0.035*
Log ΣCHL	5.23	0.743-36.8	0.097[#]
Log 4,4′-DDD	1.36	0.699-2.65	0.365
Log 4,4′-DDE	1.16	0.203-6.67	0.865
Log 4,4′-DDT	0.566	0.179-1.79	0.331
Log ΣDDT	1.32	0.185-9.36	0.783
Log Dieldrin	1.20	0.398-3.63	0.744
Log Endosulfan I	0.782	0.258-2.37	0.663
Log Endosulfan II	1.77	0.645-4.86	0.267
Log Endosulfan sulfate	0.413	0.124-1.38	0.150
Log ΣEndosulfan	0.843	0.203-3.50	0.814
Log Endrin	0.716	0.186-2.75	0.627
Log Endrin aldehyde	1.00	0.361-2.79	0.995
Log Endrin ketone	7.06	1.58-31.6	0.011*
Log ΣEndrin	2.10	0.425-10.4	0.363
Log Heptachlor	1.12	0.232-5.36	0.891
Log Heptachlor epoxide (isomer B)	0.460	0.124-1.70	0.244
Log ΣHeptachlor	0.913	0.174-4.80	0.914
Log Methoxychlor	0.875	0.288-2.66	0.814

[a]Adjusted by pre-pregnant BMI, age, population, annual income, birth year and parity.

* $p < 0.05$, [#] $p < 0.1$.

Table S18. Odds ratio of breast milk OCP residues and their associations to women with the shortest menstrual period days of ≤ 3 days as determined by logistic regression models

OCPs	Odds Ratio[a]	95% confidence intervals	*p*-value
Log Aldrin	1.15	0.275-4.83	0.845
Log α- HCH	1.41	0.297-6.65	0.668
Log β- HCH	1.05	0.143-7.68	0.963
Log γ- HCH	0.333	0.045-2.48	0.283
Log δ- HCH	0.339	0.047-2.47	0.285
Log ΣHCH	0.847	0.051-14.1	0.908
Log cis-CHL	1.27	0.201-7.98	0.801
Log trans-CHL	14.9	1.53-145	0.020*
Log ΣCHL	14.5	1.07-197	0.044*
Log 4,4′-DDD	0.936	0.448-1.96	0.861
Log 4,4′-DDE	3.33	0.532-20.9	0.199
Log 4,4′-DDT	0.741	0.219-2.51	0.630
Log ΣDDT	3.79	0.458-31.3	0.217
Log Dieldrin	0.993	0.281-3.51	0.991
Log Endosulfan I	1.89	0.500-7.12	0.349
Log Endosulfan II	1.51	0.471-4.86	0.487
Log Endosulfan sulfate	1.13	0.350-3.67	0.835
Log ΣEndosulfan	1.94	0.377-9.96	0.428
Log Endrin	0.998	0.217-4.60	0.998
Log Endrin aldehyde	0.591	0.173-2.02	0.402
Log Endrin ketone	3.60	0.718-18.1	0.119
Log ΣEndrin	1.73	0.239-12.5	0.587
Log Heptachlor	2.21	0.370-13.1	0.385
Log Heptachlor epoxide (isomer B)	1.05	0.273-4.04	0.943
Log ΣHeptachlor	2.42	0.368-16.0	0.358
Log Methoxychlor	0.609	0.161-2.30	0.464

[a] Adjusted by pre-pregnant BMI, age, population, annual income, birth year and parity.

* $p < 0.05$.

Table S19. Odds ratio of breast milk OCP residues and their associations to women who have taken contraceptives as determined by logistic regression models

OCPs	Odds Ratio[a]	95% confidence intervals	*p*-value
Log Aldrin	0.673	0.173-2.62	0.568
Log α- HCH	0.527	0.114-0.245	0.413
Log β- HCH	0.340	0.0620-1.85	0.212
Log γ- HCH	0.368	0.0430-3.16	0.362
Log δ- HCH	2.21	0.367-13.4	0.386
Log ΣHCH	0.361	0.0290-4.50	0.429
Log cis-CHL	2.19	0.371-13.0	0.386
Log trans-CHL	2.79	0.523-14.9	0.230
Log ΣCHL	4.06	0.381-43.2	0.246
Log 4,4′-DDD	0.772	0.380-1.57	0.473
Log 4,4′-DDE	0.609	0.0920-4.04	0.607
Log 4,4′-DDT	2.14	0.663-6.90	0.203
Log ΣDDT	0.826	0.108-6.30	0.853
Log Dieldrin	1.15	0.339-3.89	0.823
Log Endosulfan I	0.775	0.223-2.70	0.689
Log Endosulfan II	0.907	0.294-2.80	0.865
Log Endosulfan sulfate	0.923	0.289-2.94	0.892
Log ΣEndosulfan	0.575	0.115-2.89	0.502
Log Endrin	2.47	0.483-12.6	0.278
Log Endrin aldehyde	1.23	0.395-3.85	0.719
Log Endrin ketone	0.537	0.149-1.94	0.342
Log ΣEndrin	1.49	0.251-8.84	0.661
Log Heptachlor	0.395	0.071-2.207	0.290
Log Heptachlor epoxide (isomer B)	0.846	0.212-3.38	0.813
Log ΣHeptachlor	0.421	0.0680-2.60	0.352
Log Methoxychlor	0.587	0.173-2.00	0.394

[a]Adjusted by pre-pregnant BMI, age, population, annual income, birth year and parity.

Table S20. Odds ratio of breast milk OCP residues and their associations to women who have or have not taken hormonal drugs as determined by logistic regression models

OCPs	Odds Ratio[a]	95% confidence intervals	*p*-value
Log Aldrin	0.678	0.131-3.52	0.644
Log α- HCH	1.38	0.251-7.59	0.712
Log β- HCH	0.913	0.0900-9.27	0.938
Log γ- HCH	0.100	0.00600-1.63	0.106
Log δ- HCH	0.802	0.107-5.99	0.830
Log ΣHCH	0.660	0.0310-14.0	0.790
Log cis-CHL	0.088	0.00500-1.44	0.089
Log trans-CHL	1.62	0.308-8.51	0.569
Log ΣCHL	0.494	0.0590-4.14	0.515
Log 4,4′-DDD	1.32	0.561-3.11	0.523
Log 4,4′-DDE	0.059	0.00100-3.43	0.172
Log 4,4′-DDT	1.23	0.297-5.13	0.772
Log ΣDDT	0.0890	0.00200-4.31	0.222
Log Dieldrin	0.631	0.132-3.03	0.565
Log Endosulfan I	1.60	0.338-7.57	0.554
Log Endosulfan II	6.97	0.689-70.4	0.100[b.]
Log Endosulfan sulfate	2.48	0.540-11.34	0.243
Log ΣEndosulfan	18.6	1.22-283	0.035*
Log Endrin	0.730	0.133-4.00	0.717
Log Endrin aldehyde	0.516	0.134-1.98	0.334
Log Endrin ketone	2.37	0.431-13.1	0.321
Log ΣEndrin	1.22	0.147-10.1	0.856
Log Heptachlor	5.39	0.491-59.3	0.168
Log Heptachlor epoxide (isomer B)	16.6	1.72-160	0.015*
Log ΣHeptachlor	21.6	1.07-437	0.045*
Log Methoxychlor	0.143	0.00500-3.81	0.246

[a] Adjusted by pre-pregnant BMI, age, population, annual income, birth year and parity.

[b] $p = 0.100$ when adjusted by pre-pregnant BMI, age, population, annual income and birth year, but $p = 0.996$ if parity is also included in the adjustment.

* $p < 0.05$.

Table S21. Odds ratios of OCP residues in breast milk from mothers who received infertility medical treatment in comparison to normal mothers as determined by logistic regression models

OCPs	Odds Ratio[a]	95% confidence intervals	*p*-value
Log Aldrin	1.22	0.262–5.72	0.797
Log γ-HCH	25.6	1.26–519	0.035*
Log ΣHCH	1.45	0.091–22.9	0.793
Log ΣCHL	1.39	0.150–12.8	0.773
Log 4,4′-DDD	0.991	0.456–2.15	0.982
Log 4,4′-DDE	1.50	0.115–19.4	0.758
Log 4,4′-DDT	0.484	0.116–2.03	0.321
Log ΣDDT	1.41	0.085–23.2	0.811
Log Dieldrin	2.33	0.530–10.2	0.263
Log ΣEndosulfan	1.14	0.201–6.43	0.885
Log ΣEndrin	0.346	0.026–4.60	0.421
Log ΣHeptachlor	1.63	0.237–11.2	0.620
Log Methoxychlor	0.763	0.184–3.17	0.710

[a] Adjusted by pre-pregnant BMI, age, population, annual income, birth year and parity.

* $p < 0.05$.

Table S22. Odds ratios of breast milk OCP residues in the participants having undergone gynecological surgery compared with those in normal women as determined by logistic regression models

	Odds ratio (*p*-value)	Odds ratio[a] (*p*-value)	Odds ratio[b] (*p*-value)
Gynecological surgery			
Log ΣHCH	0.248 (0.332)	0.129 (0.344)	0.203 (0.481)
Log ΣCHL	0.664 (0.705)	0.838 (0.892)	0.577 (0.733)
Log ΣDDT	0.790 (0.849)	1.61 (0.756)	2.24 (0.631)
Log ΣEndosulfan	0.630 (0.594)	1.98 (0.519)	1.52 (0.724)
Log ΣEndrin	0.209 (0.209)	0.330 (0.511)	0.130 (0.301)
Log ΣHeptachlor	0.367 (0.272)	0.797 (0.843)	1.17 (0.900)

[a] Adjusted by pre-pregnant BMI, age, population and annual income.

[b] Adjusted by pre-pregnant BMI, age, population, annual income, birth year and parity.

Table S23. Sociodemographic characteristics, dietary habits and menstruation characteristics of mothers in association with OCP residues in breast milk as determined using the logistic regression model

	Odds Ratio[a]	95% confidence intervals	*p*-value
Population (native-born Aborigines)[b]			
Log Aldrin	5.63[c]	0.852-37.2	0.073
Log trans-CHL	6.48[c]	0.890-47.1	0.065
Log ΣCHL	13.2[c]	0.706-246	0.084
Log Endosulfan sulfate	10.8[c]	1.03-113	0.047*
Pre-pregnant BMI values (>21.7 kg m^{-2})[b]			
Log 4,4′-DDT	0.478[d]	0.201-1.14	0.095
Log Heptachlor	2.81[d]	0.924-8.56	0.069
Annual family income (≤$20,000 US dollar)[b]			
Log α- HCH	4.20[e]	1.08-16.2	0.037*
Log ΣHCH	10.7[e]	1.27-90.1	0.029*
Gravidity (primiparous)[b]			
Log β- HCH	5.14[f]	0.884-29.9	0.068
Log ΣHCH	27.3[f]	1.63-457	0.021*
Log Dieldrin	0.199[f]	0.038-1.04	0.056
Cow milk consumption (>625 mL $week^{-1}$)[b]			
Log Aldrin	2.73	0.843-8.83	0.094
Log β- HCH	7.35	1.45-37.3	0.016*
Log ΣHCH	7.35	0.894-60.3	0.064
Log ΣCHL	6.65	1.10-40.1	0.039*
Log Endosulfan I	3.03	1.04-8.83	0.043*
Log Endosulfan II	4.53	1.56-13.1	0.005**
Log ΣEndosulfan	6.67	1.53-28.9	0.011*
Log Endrin	6.73	1.32-34.4	0.022*
Log Endrin ketone	3.72	1.18-11.7	0.025*
Log ΣEndrin	13.3	2.05-86.1	0.007**
Log ΣHeptachlor	9.11	1.72-48.4	0.010*
Beef consumption (>50 g $week^{-1}$)[b]			
Log γ- HCH	0.128	0.017-0.946	0.044*
Log trans-CHL	5.05	1.25-20.4	0.023*
Log ΣCHL	10.5	1.36-81.2	0.024*
Log Endosulfan II	4.18	1.27-13.8	0.019*
Log Endosulfan sulfate	7.16	1.36-37.7	0.020*
Log ΣEndosulfan	6.88	1.24-38.0	0.027*
Log Endrin ketone	3.68	0.957-14.2	0.058
Log Methoxychlor	4.01	1.13-14.2	0.032*
Average periods of menstrual cycle ≤ 26 and ≥ 30 days[b]			
Log Endosulfan II	0.409	0.157-1.07	0.067
Average menstrual period days (>5 days)[b]			
Log trans-CHL	4.73	1.12-20.1	0.035*
Log ΣCHL	5.23	0.743-36.8	0.097
Log Endrin ketone	7.06	1.58-31.6	0.011*
Shortest menstrual period days (≤ 3 days)[b]			
Log trans-CHL	14.9	1.53-145	0.020*
Log ΣCHL	14.5	1.07-197	0.044*
Have taken hormonal drugs[b]			
Log ΣEndosulfan	18.6	1.22-283	0.035*
Log Heptachlor epoxide (isomer B)	16.6	1.72-160	0.015*
Log ΣHeptachlor	21.6	1.07-437	0.045*
Infertility[b]			
Log γ-HCH	25.6	1.26–519	0.035*

[a]Adjusted for pre-pregnant BMI, age, population, annual income, birth year, and parity.

[b] The reference groups are native-born and nonnative-born Taiwanese, lower annual family income (<$ 20,000 US dollars), multiparous mothers, women having lower consumption of cow milk (<625 mL $week^{-1}$), mothers having lower consumption of beef (<50 g $week^{-1}$), women with normal period of averaged menstrual period, women with normal period of the shortest menstrual period, mothers without taking hormonal drugs, and women without experience with infertility treatment.

[c]Adjusted by pre-pregnant BMI, age, annual income, birth year and parity.
[d] Adjusted by age, population, annual income, birth year and parity.
[e] Adjusted by pre-pregnant BMI, age, population, birth year and parity.
[f] Adjusted by pre-pregnant BMI, age, population, annual income, and birth year.
* $p < 0.05$, ** $p < 0.01$,*** $p < 0.001$.

Limitation of small sample size

As stated in the literature review of this manuscript, Bastos et al. (2013) indicated that women receiving infertility treatment had the higher serum DDE compared to those who were spontaneously pregnant in Brazil (n = 36). Rojas-Squella et al. (2013) recruited 32 women in a Colombian study and detected 4,4′DDE with concentrations ranging from <25 to 14,948 ng/g; moreover, significant changes in the pesticide concentration was observed during lactation. Chao et al., 2006 also used a small sample size (n = 36) for the investigation of human milk OCP concentrations in Central Taiwan. More recently, Polanco Rodriguez et al. (2017) obtained 24 breast milk samples from Maya women in rural areas of Yucatan, Mexico and they observed that high levels of OCPs have possible associations to the high rates of cervical uterine and breast cancer mortality in those areas. Accordingly, the sample sizes of these papers are smaller than that of this study, although the significant findings of our present study are also limited by the small sample size.

References:

Rojas-Squella, X.; Santos, L.; Baumann, W.; Landaeta, D.; Jaimes, A.; Correa, J. -C.; Sarmiento, O. -L.; Ramos-Bonilla, J. -P., Presence of organochlorine pesticides in breast milk samples from Colombian women. *Chemosphere* **2013**, 91, (6), 733-739.

Polanco Rodriguez, A.G., Inmaculada Riba Lopez, M., Angel DelValls Casillas, T., Leon, J.A., Anjan Kumar Prusty, B. and Alvarez Cervera, F.J. (2017) Levels of persistent organic pollutants in breast milk of Maya women in Yucatan, Mexico. Environ Monit Assess 189(2), 59.

Bastos, A. M. X.; Souza, M. d. C. B. d.; Almeida Filho, G. L. d.; Krauss, T. M.; Pavesi, T.; Silva, L. E. d., Organochlorine compound levels in fertile and infertile women from Rio de Janeiro, Brazil. *Arquivos Brasileiros de Endocrinologia & Metabologia* **2013,** 57, (5), 346-353.

Chao, H.-R.; Wang, S.-L.; Lin, T.-C.; Chung, X.-H., Levels of organochlorine pesticides in human milk from central Taiwan. *Chemosphere* **2006,** 62, (11), 1774-1785.
